# Temperature Dramatically Shapes Mosquito Gene Expression With Consequences for Mosquito–Zika Virus Interactions

**DOI:** 10.3389/fmicb.2020.00901

**Published:** 2020-06-12

**Authors:** Priscila Gonçalves Ferreira, Blanka Tesla, Elvira Cynthia Alves Horácio, Laila Alves Nahum, Melinda Ann Brindley, Tiago Antônio de Oliveira Mendes, Courtney Cuinn Murdock

**Affiliations:** ^1^Department of Biochemistry and Molecular Biology, Universidade Federal de Viçosa, Viçosa, Brazil; ^2^Department of Infectious Diseases, College of Veterinary Medicine, University of Georgia, Athens, GA, United States; ^3^René Rachou Institute, Oswaldo Cruz Foundation, Belo Horizonte, Brazil; ^4^Department of Genetics, Ecology and Evolution, Institute of Biological Sciences, Federal University of Minas Gerais, Belo Horizonte, Brazil; ^5^Promove College of Technology, Belo Horizonte, Brazil; ^6^Department of Population Health, College of Veterinary Medicine, University of Georgia, Athens, GA, United States; ^7^Center for Vaccines and Immunology, University of Georgia, Athens, GA, United States; ^8^Odum School of Ecology, University of Georgia, Athens, GA, United States; ^9^Center for the Ecology of Infectious Diseases, University of Georgia, Athens, GA, United States; ^10^Center for Emerging and Global Tropical Diseases, University of Georgia, Athens, GA, United States; ^11^River Basin Center, University of Georgia, Athens, GA, United States; ^12^Department of Entomology, College of Agriculture and Life Sciences, Cornell University, Ithaca, NY, United States

**Keywords:** temperature, *Aedes aegypti*, Zika virus, RNA-seq, transcriptome, immune response

## Abstract

Vector-borne flaviviruses are emerging threats to human health. For successful transmission, the virus needs to efficiently enter mosquito cells and replicate within and escape several tissue barriers while mosquitoes elicit major transcriptional responses to flavivirus infection. This process will be affected not only by the specific mosquito-pathogen pairing but also by variation in key environmental variables such as temperature. Thus far, few studies have examined the molecular responses triggered by temperature and how these responses modify infection outcomes, despite substantial evidence showing strong relationships between temperature and transmission in a diversity of systems. To define the host transcriptional changes associated with temperature variation during the early infection process, we compared the transcriptome of mosquito midgut samples from mosquitoes exposed to Zika virus (ZIKV) and non-exposed mosquitoes housed at three different temperatures (20, 28, and 36°C). While the high-temperature samples did not show significant changes from those with standard rearing conditions (28°C) 48 h post-exposure, the transcriptome profile of mosquitoes housed at 20°C was dramatically different. The expression of genes most altered by the cooler temperature involved aspects of blood-meal digestion, ROS metabolism, and mosquito innate immunity. Further, we did not find significant differences in the viral RNA copy number between 24 and 48 h post-exposure at 20°C, suggesting that ZIKV replication is limited by cold-induced changes to the mosquito midgut environment. In ZIKV-exposed mosquitoes, vitellogenin, a lipid carrier protein, was most up-regulated at 20°C. Our results provide a deeper understanding of the temperature-triggered transcriptional changes in *Aedes aegypti* and can be used to further define the molecular mechanisms driven by environmental temperature variation.

## Introduction

Over the past three decades, there have been significant advances in our understanding of the physiological and molecular interactions between pathogens and mosquito vectors (Bartholomay and Michel, 2018; Kumar et al., 2018; Simões et al., 2018). Research has provided substantial insights into the immune genes and pathways that shape resistance to vector-borne pathogens and has revealed many promising targets for genetic manipulation (Wilke and Marrelli, 2015; Yen et al., 2018; Shaw and Catteruccia, 2019). However, we are only just beginning to understand the complexity underlying mosquito–pathogen interactions. The response mosquitoes mount toward a given pathogen is a dynamic phenotype that is dependent upon both the specific mosquito–pathogen pairing (Lambrechts et al., 2013; Zouache et al., 2014; Duggal et al., 2015; Chouin-Carneiro et al., 2016) and the variation in key environmental factors (Okech et al., 2007; Alto et al., 2008; Parham and Michael, 2010; Parham et al., 2015; Cohen et al., 2016; Gloria-Soria et al., 2017; Murdock et al., 2017; Shragai et al., 2017; Siraj et al., 2018). Knowledge of how vectors respond to environmental variation is especially relevant for understanding how vector-borne pathogens emerge, defining the biological constraints on transmission, and anticipating the robustness of novel vector-control approaches (that manipulate mosquito physiological and immunological mechanisms to limit virus infection) in field settings.

Vector-borne flaviviruses are emerging threats to human health. Yellow fever virus (YFV), dengue virus (DENV), West Nile virus (WNV), and most recently, Zika virus (ZIKV) can be found throughout tropical and subtropical zones. For successful transmission, flaviviruses are taken up through the bite of a mosquito vector when it takes a blood meal from an infectious host. To complete infection within the mosquito vector, the virus needs to efficiently enter, replicate within, and escape several tissue barriers, primarily the midgut and salivary glands (Franz et al., 2015; Kumar et al., 2018). A number of studies have demonstrated that mosquitoes elicit major transcriptional changes in response to flavivirus infection, which could play an important role in limiting infection (Xi et al., 2008; Sim and Dimopoulos, 2010; Colpitts et al., 2011; Bonizzoni et al., 2012; Chauhan et al., 2012; Angleró-Rodríguez et al., 2017; Etebari et al., 2017; Saldaña et al., 2017a). These include differential regulation of genes involved in RNA interference (RNAi), classical immune pathways (e.g., JAK-STAT, Toll), production and transport of energy, and metabolism, as well as the production of non-coding RNAs (small and long) and microRNAs that could be involved in targeted gene regulation. Currently, it is unclear how conserved these responses to infection are across different mosquito–flavivirus combinations (Etebari et al., 2017) and how relevant environmental variation shapes the nature, magnitude, and timing of these responses (Murdock et al., 2012b, 2014; Adelman et al., 2013).

One of the major environmental variables that influence mosquito–pathogen interactions, as well as vector-borne transmission, is variation in environmental temperature. Mosquitoes are small ectothermic organisms, and many studies have already demonstrated that temperature can markedly affect diverse aspects of mosquito physiology, ecology, and pathogen replication. This, in turn, will shape the proportion of mosquitoes that become infected and infectious, the overall transmission potential of mosquito populations (Christofferson and Mores, 2016; Murdock et al., 2017; Tesla et al., 2018a; Mordecai et al., 2019), and how the distributions of mosquito vectors and vector-borne pathogens will shift in response to climate change (Siraj et al., 2014; Campbell et al., 2015; Ryan et al., 2015, 2018; Misslin et al., 2016). The extent to which temperature shapes transmission directly, through effects on pathogen biology, or indirectly, through effects on mosquito immunity and physiology, remains largely unexplored (Murdock et al., 2012a).

**FIGURE 1:**
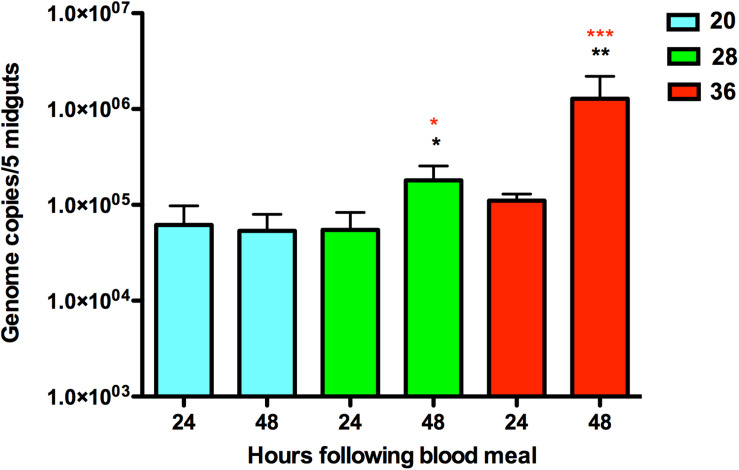
Quantification of ZIKV RNA in infected *Aedes aegypti* mosquitoes housed at 20, 28, and 36°C at two time points, 24 and 48 h. The average number of ZIKV genome copies present in pools of five midgut samples and standard error are shown. The *y*-axis is log-transformed. Black asterisks represent *p*-value < 0.05 calculated by *T*-test between the time points of 24 and 48 h post-infection for the same temperature. Red asterisks represent *p*-value < 0.05 calculated by ANOVA with Tukey correction for multiple hypotheses between the three different temperatures for the same time point after blood meal.

**FIGURE 2:**
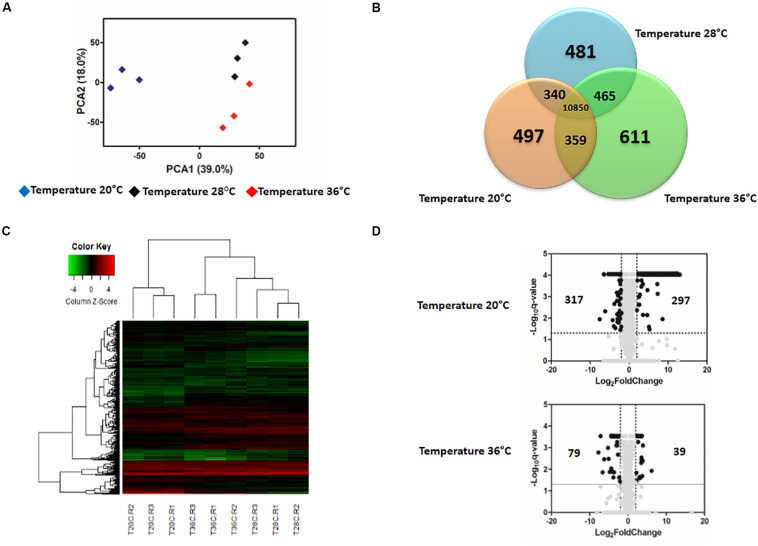
Effect of temperature in the expression profile of uninfected blood-fed *Aedes aegypti.* **(A)** Principal Component Analysis (PCA) plot showing the global gene expression profiles. **(B)** Venn Diagram reporting the number of specific and shared genes. **(C)** Heatmap plot showing local differences (naming scheme: TC20R1 = replicate 1 for the temperature 20°C). **(D)** Volcano plot representing the differential gene expression in RNAseq samples from *Ae. aegypti* exposed to three different constant temperatures (20, 28, and 36°C) at 48 h.

In previous work (Tesla et al., 2018a), we have demonstrated that ZIKV transmission by *Aedes aegypti* is optimized at a mean temperature of 29°C and has a thermal range from 22.7 to 34.7°C. Further, we observed constraints on ZIKV transmission at cool and warm temperatures to be mediated by different factors. To overcome the midgut barrier, a minimal viral load is necessary (Vazeille et al., 2019). Cool temperatures inhibited ZIKV transmission due to slow virus replication and escape from the midgut, resulting in a decrease in metrics of vector competence (e.g., the proportion of mosquitoes that became infected, disseminated infection, and became infectious) and lengthening the extrinsic incubation period (e.g., the interval of time before ZIKV can be detected in the saliva and mosquitoes become infectious [Tesla et al., 2018a; see Figures 1, 2)]. In contrast, increased mosquito mortality at hotter temperatures constrained transmission despite efficient ZIKV infection and rapid dissemination.

These temperature constraints on infection are likely regulated through different mechanisms. Temperature variation in general could profoundly impact arbovirus infection and replication early in infection due to shifts in the balance and dynamics of the midgut environment, the first host environment encountered. This environment is fairly complex, as arboviruses encounter oxidative and nitration stress associated with digestion of the blood meal (Luckhart et al., 1998; Graça-Souza et al., 2006; Xi et al., 2008), the presence [e.g., *Wolbachia* (Bian et al., 2010)] and proliferation of microbial flora (Xi et al., 2008; Carissimo et al., 2014; Hegde et al., 2015; Saraiva et al., 2016; Barletta et al., 2017; Saldaña et al., 2017b), and key immune factors (Campbell et al., 2008; Xi et al., 2008; Cirimotich et al., 2009; Sánchez-Vargas et al., 2009; Souza-Neto et al., 2009). To better understand the effects of temperature on the ZIKV-mosquito interaction, we used RNA sequencing to describe the transcriptional response of *Ae. aegypti* midguts to ZIKV during the early infection process at three different temperatures (20, 28, and 36°C) previously shown to impact ZIKV infection, dissemination, and transmission rates (Tesla et al., 2018a).

## Materials and Methods

### Ethics Statement

All mosquito infection work with ZIKV was approved by the University of Georgia, Athens Institutional Biosafety Committee (reference number 2015-0039).

### Virus Production

Zika virus MEX1-44 was isolated from *Ae. aegypti* mosquitoes from Tapachula, Chiapas, Mexico in January 2016, kindly provided by the University of Texas Medical Branch Arbovirus Reference Collection. ZIKV stocks were propagated in Vero cells cultured in DMEM (Dulbeco’s Modified Eagle Medium), 5% fetal bovine serum (FBS) at 37°C and 5% CO_2_. Four days following inoculation, when cells showed visible cytopathic effect (>90%), supernatant containing virus was collected, cell debris was cleared by centrifugation (3,500 × *g* for 5 min), and virus was aliquoted and frozen at −80°C. The stock was titrated using standard plaque assays on Vero cells (Willard et al., 2017) and expressed in plaque-forming units per milliliter (PFU/mL).

### Mosquito Husbandry

*Aedes aegypti* eggs collected in Chiapas, Mexico, were hatched in ddH_2_O under reduced pressure in a vacuum desiccator. Larvae were grown in trays, with 200 larvae in 1L ddH_2_O and four fish food pellets (Hikari Cichlid Cod Fish Pellets). Emerging adults were kept in rearing cages and fed with 10% sucrose solution *ad libitum*. Colonies were maintained on O-positive human blood (Interstate Blood Bank, male donors between 30 and 35 year). Both larvae and adults were maintained under the standard insectary conditions (27°C ± 0.5°C, 80% ± 5% relative humidity, and a 12:12 light: dark diurnal cycle) (Percival Scientific) that replicate rearing and experimental conditions used in Tesla et al. (2018a).

### Experimental Mosquito Infection

Briefly, 3 to 4-day-old female mosquitoes (F5 generation; *n* = 600) were separated using a vacuum aspirator, transferred to an arthropod containment level three (ACL-3) facility at the University of Georgia and housed at 28°C ± 0.5°C, 80% ± 5% relative humidity, 12 h:12 h light:dark cycle (Percival Scientific). Mosquitoes were fed with a non-infectious or ZIKV-containing blood-meal (10^6^ PFU/mL) after a 12-h period of starvation in a manner previously described (Tesla et al., 2018b). After the feed, engorged ZIKV-exposed (*n* = 120) and unexposed mosquitoes (*n* = 120) were randomly allocated across 12 paper cups (six with ZIKV-exposed and six with ZIKV-unexposed mosquitoes, *n* = 20 mosquitoes per cup). Forty engorged females that fed on either infectious blood or non-infectious blood were randomly distributed across one of three temperature treatments, 20°C, 28°C, and 36°C (±0.5°C) at 80% ± 5% relative humidity and with a 12 h:12 h light:dark cycle. Mosquitoes were provided 10% sucrose *ad libitum* throughout the duration of the experiment (48 h), and three full biological replicates were performed.

### Viral Genome Quantification

Viral genomes were detected as previously described (Willard et al., 2019). Briefly, dissected mosquito midguts were collected in pools of five, and viral RNA was isolated using the Zymo Quick-RNA Viral Kit (Zymo, Irvine, CA, United States). Viral RNA samples were reverse-transcribed (RT) to cDNA (High Capacity RNA-to-cDNA Kit, Applied Biosystems, Foster City, CA, United States). The F5 generation was used to maintain genetic proximity with field populations of *Ae. aegypti*. To quantify the number of ZIKV genomes, we used the cDNA in a quantitative PCR (qPCR) reaction assay using TaqMan Gene Expression Master Mix (Applied Biosystems, ThermoFisher, Waltham, MA, United States), primers, and probes (F: ZIKV 1086, R: ZIKV 1162c, ZIKV 1107-FAM; TaqMan MGB Probe; Invitrogen Custom Primers) (Lanciotti et al., 2008). Each sample was analyzed in duplicate, and each plate contained a DNA plasmid standard curve (ZIKV molecular clone), no template, and no primer controls. ZIKV copy numbers were extrapolated from the generated standard curve using the Applied Biosystems protocol. The limit of detection was experimentally established to be 30 copies. Final copy numbers were adjusted by back-calculations to the total RNA and cDNA volume and expressed as copies per five-midgut pool. Outliers were identified by Grubbs test, implemented in Graphpad QuickCalcs^1^, and removed. The results were analyzed in GraphPad Prism 5.0. An unpaired *T*-test was applied to compare the two different times (24 and 48) post-infection for the same temperature. ANOVA with Tukey as the multiple hypothesis correction was used to compare the data from the three different temperatures (20, 28, and 36) at the same time points. A *p*-value below 0.05 was considered statistically significant.

### Midgut Dissection and RNA Isolation

Mosquito responses to blood meal digestion, midgut microbial proliferation, and arbovirus midgut infection contribute significantly to viral bottlenecks during infection, and these processes could dynamically differ with temperature (Xi et al., 2008; Bonizzoni et al., 2011; Oliveira et al., 2011; Ramirez et al., 2012). Further, these responses likely occur early on during the infection process (<3 days post-infection) at optimal temperatures for ZIKV transmission (e.g., 29°C), as the proportion of mosquitoes with ZIKV-infected midguts plateaus by day three post-infection at temperatures above 28°C (Tesla et al., 2018a). To capture gene expression changes in the mosquito midgut early during the infection process, we isolated total RNA from midgut tissues. Fifteen ZIKV-exposed and 15 non-exposed mosquitoes were removed from each temperature at 24 and 48 h post-infection for RNA sequencing. The mosquitoes were killed with cold ethanol and washed in cold PBS containing 0.2% RNase inhibitor (Sigma-Aldrich) and 0.1% DEPC (Amresco). Midguts were carefully dissected and stored immediately in RNAlater (Invitrogen) at 4°C for 24 h, after which they were transferred to −80°C conditions. Total RNA was isolated from pools of 15 midguts using the Qiagen RNeasy Mini Kit as per the manufacturer’s instructions.

### Library Preparation and Sequencing

Extracted RNA was sent to the University of Georgia Genomics Core for cDNA library preparation and RNA sequencing. The quality of the total RNA was analyzed using an Agilent Bioanalyzer RNA 6000 Pico Kit (Agilent Technologies). Poly(A) mRNA from total RNA was captured by magnetic oligo-dT beads and fragmented by heating (94°C, 6 min) in the presence of divalent cations (Mg^2+^) using the KAPA Stranded mRNA-Seq Kit for Illumina^®^. Fragmented poly(A) mRNA samples were converted to cDNA using random priming. After second-strand synthesis, an adenine residue was added to 3′-end of dscDNA fragments, and Illumina TruSeqLT adapter ligation to 3′-dAMP library fragments was performed. Adapter-ligated DNA was amplified by PCR using the KAPA Library Amplification Primer Mix. After creating the library of DNA templates, the fragment size distribution of these libraries was confirmed by electrophoresis and the library concentration was determined by qPCR. Sequencing was completed on an Illumina NextSeq 500 using the PE75bp (75 bp sequencing reads) settings on a High-Output 150-cycle kit using Illumina standard protocols for loading. Raw sequencing files were demultiplexed using the Illumina BaseSpace cloud platform demultiplexing pipeline. Four technical replicates were run per sample.

### RNA Sequence Analysis in Response to Temperature and Zika Virus Exposure

The Tuxedo suite of tools was used to analyze the RNA-seq data (Trapnell et al., 2013). At each stage of the analysis pipeline, we were careful to identify and correct possible sources of bias in the study (Conesa et al., 2016). Read quality was assessed using FastQC (Andrews, 2010). Poor quality reads (quality score below 20), short reads (less than 25 bases), and adapter sequences (Bolger et al., 2014) were removed using Trimmomatic (v 0.36).

We aligned and mapped clean reads up to two different loci to the *Ae. aegypti* genome (NCBI ID: GCA_002204515.1) obtained from NCBI^2^ using TopHat (v 2.1.1) (Trapnell et al., 2009). We then performed differential gene expression analysis on RNAseq reads using Cuffdiff (Trapnell et al., 2013), which calculates expression levels in response to experimental treatments of interest (e.g., temperature and ZIKV-exposure) and determines whether observed variations in expression levels across groups are significantly different. The *Ae. aegypti* genome and its annotation file (NCBI ID: GCA_002204515.1) were used to run bias detection and determine the transcripts examined for differential expression, respectively. The relative expression levels were produced as fragments per kilobase of transcript per million fragments mapped (FPKM values), which normalized read count based on gene length and the total number of mapped reads. After quantification and normalization of expression values, differential expression analysis was carried out on the experimental data from 24 and 48 h following the blood meal. To characterize changes in the RNA transcriptome in response to temperature, we compared the RNA expression profiles of unexposed mosquitoes maintained at standard conditions and near the predicted thermal optimum for ZIKV transmission (28°C) to those of mosquitoes housed under cool (20°C) and hot (36°C) conditions. To determine whether temperature modified global RNA expression in ZIKV-exposed individuals, we compared whether the variation in expression profiles of ZIKV-exposed individuals across temperature treatments was similar to that of their unexposed counterparts. Finally, to evaluate the effects of ZIKV infection on global RNA expression, we compared RNA expression between unexposed and ZIKV-exposed mosquitoes within a given temperature treatment. The data quality assessment from the RNAseq analysis was performed using R package (v 3.4.4) (R Development Core Team, 2008) and plotted using GraphPad Prism (version 5.01 for Windows, GraphPad Software, San Diego, CA, United States)^3^. Perl scripts were written to extract specific information from RNAseq analysis output files necessary for each assessment of the results obtained. Principal-component analysis (PCA) was employed to examine the quality of replicates and the overall differences between samples and to determine the proximity among the experimental groups.

### Gene Ontology (GO) Analysis

We employed BiNGO (v 3.0.3) (Maere et al., 2005) to determine which GO terms were significantly over-represented in a set of genes. First, the FASTA sequences of all differentially expressed genes were recovered using the protein database on the NCBI’s Batch Entrez^4^. The sequences were inputted into STRING (v 10.5) (Szklarczyk et al., 2017) to predict protein–protein interactions. GO functional annotations were provided by AgBase-Goanna (v 2.00) (Mccarthy et al., 2006), using the *Ae. aegypti* protein sequences in FASTA format from the STRING database as an input file. The similarity search for GO annotations was performed by Blastp using the UniProt Database with *E*-value: 10e^–5^, Matrix: BLOSUM62, and word size: 3. This file annotation was employed as a reference set for enrichment analysis. We used the default mode, employing Hypergeometric tests for assessing the enrichment of a GO term in the test set, and *p*-values were corrected by Benjamini and Hochberg False Discovery Rate (FDR) correction to control for type I error. Corrected *p*-values less than 0.05 were considered significant.

## Results

From a total of 36 RNA samples that were sequenced using the Illumina platform, the number of clean paired reads varied between 2,063,917 and 3,028,305 for each library. All libraries resulted in a concordant pair alignment rate higher than 70%.

### Cool Temperatures Restrict ZIKV Replication in the Mosquito Midgut

Viral RNA quantification of exposed mosquitoes revealed levels of ZIKV RNA in midgut samples from mosquitoes that imbibed a blood meal containing ZIKV (Figure 1). ZIKV replication was evident in mosquitoes housed at 28°C and 36°C, with increases in mosquito RNA copy number between 24 and 48 h post-feed (hpf). Further, the efficiency of ZIKV replication was maximized at the warmest temperature, with mosquitoes housed at 36°C having higher ZIKV RNA levels in their midguts than those housed at 28°C. Although the presence of ZIKV RNA was observed 24 and 48 h following the infectious blood meal in mosquitoes housed in the cool environment (20°C), we did not find significant increases in the viral copy number (Figure 1). This result suggests that cooler temperatures constrain ZIKV replication in the midgut.

### The Effect of Temperature on Gene Expression in Unexposed Mosquitoes

Gene-expression patterns were influenced by both temperature and time post-blood feed. In general, the principal component analysis (PCA) plot showed a high degree of reproducibility among the replicate samples within each temperature treatment. At 24 hpf, temperature clearly distinguished gene expression profiles (Supplementary Figure S1A). However, by 48 hpf, mosquitoes housed at 28°C and 36°C had gene expression profiles that were more similar to each other than to mosquitoes housed at 20°C (Figure 2A). According to the Venn diagram, the vast majority of genes expressed in the midgut were similar for mosquitoes housed across the three temperature treatments (10,459 at 24 hpf, Supplementary Figure S1B; 10,850 at 48 hpf, Figure 2B). When concentrating on the differentially expressed genes, Euclidean distance heatmap analysis demonstrated that samples at 24 hpf were separated by temperature treatment in three distinct groups (Supplementary Figure S1C), while for samples at 48 hpf, 28 and 36°C samples cluster more closely with one another than with the 20°C (Figure 2C). This is the same profile as was seen in the PCA analysis, which employed all transcriptome data. Among these genes, 1665 differentially expressed genes (*q*-value < 0.05) were seen between mosquitoes held at 20 and 28°C for 24 h, and 3634 were seen by 48 h (Supplementary Table S1). In contrast, when comparing mosquitoes held at 36 and 28°C, we identified 1697 and 1695 differentially expressed genes after 24 and 48 h, respectively (Supplementary Table S2). In order to perform a more stringent analysis, we produced a volcano plot, which indicated a total of 70 and 297 upregulated genes [log_2_(Fold Change) >2 and *q*-value < 0.05] in mosquitoes housed at 20°C for 24 and 48 h, respectively, compared to those housed at 28°C. For downregulated genes [log_2_(Fold Change) <−2 and *q*-value < 0.05], we found 53 and 317 after 24 and 48 h, respectively (Supplementary Figure S1D and Figure 2D). We did not find as strong an effect of exposure time when mosquitoes were housed at 36°C, with only 19 and 39 genes up-regulated and 89 and 79 genes down-regulated after 24 and 48 h, respectively (Supplementary Figure S1D and Figure 2D).

To further define the effect of temperature on mosquito cellular and physiological responses, we sorted the top 20 up- or down-regulated genes at both the cool and hot temperatures, using mosquitoes maintained at 28°C as our standard (Table 1). The cool temperature produced a larger response, up-regulating genes hundreds of times compared to standard conditions, whereas the 36°C treatment had a more moderate effect. The transcript with the highest enrichment at 20°C was protein-G12 (1459-fold), whereas a serine protease easter was the most down-regulated (187-fold). For mosquitoes housed at 36°C, heat shock protein 70 (HSP 70) was the transcript most enriched (71-fold) relative to 28°C, while facilitated trehalose transporter Tret1 was the most down-regulated gene (212-fold). Surprisingly, both cool and warm temperature treatments induced some of the same genes to be differentially regulated. Six genes (protein G12, serine protease SP24D, and chymotrypsin-2) were up-regulated in mosquitoes housed at both 20°C and 36°C when compared to those at 28°C, while six genes (serine protease easter, facilitated trehalose transporter Tret1, venom protease, solute carrier family 2 facilitated glucose transporter member 3, and tryptase) were down-regulated.

**TABLE 1:** Top 20 up- and down-regulated genes of uninfected *Aedes aegypti* in response to low (20°C) and to high (36°C) temperature for 48 h post-blood-feeding (relative to standard rearing temperature of 28°C).

GENES UP-REGULATED		TEMPERATURE 20°C		TEMPERATURE 36°C	
Gene ID	Gene Description	Fold Change	*q*-value	Fold Change	*q*-value
XP_021701760.1	Protein G12	1459.541989	0.0000886602	11.11657146	0.000290131
XP_021698904.1	Chymotrypsin-2	857.284352	0.0000886602		
XP_001660827.1	Protein G12	603.3619885	0.0000886602	7.4143488679	0.000290131
XP_001650490.2	Alpha-N-acetylgalactosaminidase	548.7746389	0.0000886602		
XP_001659962.1	Serine protease SP24D	473.2635493	0.0000886602	11.29286782	0.000290131
XP_021712126.1	Protein G12-like	442.0421098	0.0000886602		
XP_021701761.1	Protein G12	402.0329675	0.0000886602	6.3523041907	0.000548702
XP_001663002.1	Trypsin 3A1-like	384.5293743	0.0110252		
XP_021705369.1	Beta-galactosidase	315.6714553	0.0000886602		
XP_001656375.1	Protein G12	245.4048583	0.0000886602		
XP_001656377.1	Protein G12 isoform X2	221.8114454	0.0000886602	5.0806039301	0.000290131
XP_021703511.1	Lysosomal alpha-mannosidase isoform X2	217.023882	0.0000886602		
XP_001647937.2	Phosphoenolpyruvate carboxykinase [GTP]	180.2043466	0.0000886602		
XP_001659796.1	Beta-1,3-glucan-binding protein	159.2092116	0.000730421		
XP_021712858.1	Protein G12 isoform X2	149.8743826	0.000256108		
XP_001657509.1	Vitellogenin-A1-like	138.4047839	0.0000886602		
XP_001659961.1	Chymotrypsin-2	110.7367288	0.0000886602	8.596677982	0.000290131
XP_001652194.1	Protein singed wings 2	84.66536466	0.0000886602		
XP_001660818.2	Vitellogenin-A1	67.79655212	0.0000886602		
XP_001654186.1	Glutamine synthetase 1 mitochondrial	65.69082595	0.0000886602		
XP_021693649.1	Heat shock protein 70 A1			71.53314665	0.0118095
XP_001660673.2	Trypsin 5G1-like			14.98866514	0.0007852
XP_001663776.1	Protein G12			14.35856831	0.00416417
XP_001658359.2	Brachyurin			12.24869336	0.000290131
XP_021701762.1	Protein G12-like			12.07440339	0.00379042
XP_001663895.2	Trypsin 5G1			11.04851064	0.00505811
XP_001658471.2	Mite group 2 allergen Gly			10.38200486	0,000290131
XP_011493274.2	Extensin			9.222769233	0.000290131
XP_001659492.2	Serine protease SP24D			7.5082672369	0.000290131
XP_001661186.2	Protein G2			6.9220050786	0.000290131
XP_001651623.2	Surface antigen CRP170			6.151678044	0.000290131
XP-021712126.1	Protein G12-like			5.6022641153	0.000548702
XP_001660908.1	Maltase 1			5.3506716535	0.000290131
XP_011492940.1	Peptidoglycan-recognition protein SC-2			5.030075309	0.000290131

**GENES DOWN-REGULATED**		**TEMPERATURE 20°C**		**TEMPERATURE 36°C**	
**Gene ID**	**Gene Description**	**Fold Change**	***q*-value**	**Fold Change**	***q*-value**

XP_001652078.2	Serine protease easter	187.8360285	0.0112238	33.06859855	0.0032544
XP_001658147.1	Facilitated trehalose transporter Tret1	39.72595509	0.0000886602	212.5163457	0.00166701
XP_001652075.2	Venom protease	31.87315659	0.0000886602	21.42212957	0.000290131
XP_001663064.2	UDP-glucuronosyltransferase 2B18	28.70798168	0.0000886602		
XP_001652079.1	Serine protease easter	18.68203877	0.0124204	15.08894466	0.0134155
XP_001655069.1	Queuine tRNA-ribosyltransferase accessory subunit 2	15.34875785	0.0000886602		
XP_001656516.2	Xaa-Pro aminopeptidase ApepP	15.23840501	0.0000886602		
XP_001649987.2	Synaptic vesicle glycoprotein 2B	13.81630566	0.00215644		
XP_021706766.1	Glycerol-3-phosphate dehydrogenase mitochondrial isoform X1	13.39803945	0.0000886602		
XP_021699787.1	Solute carrier family 2 facilitated glucose transporter member 3	13.34678167	0.0000886602	99.27028299	0.0138613
XP_001655305.2	Protein MAK16 homolog A	12.43254717	0.0000886602		
XP_001649768.1	Exosome complex component RRP43	11.98593043	0.0000886602		
XP_001653029.1	Protein takeout	11.66609644	0.0000886602		
XP_021698953.1	CAD protein	11.58599466	0.0291848		
XP_001656680.1	NHP2-like protein 1 homolog	10.79948043	0.0000886602		
XP_001655729.2	Tryptase	10.69882162	0.0000886602	13.05191869	0.000290131
XP_001662512.1	Nucleoside diphosphate kinase	10.5849313	0.0000886602		
XP_001663497.1	Protein lethal(2)essential for life	10.23543137	0.0000886602		
XP_001655105.1	Sorbitol dehydrogenase	10.21211653	0.0000886602		
XP_021699336.1	Nucleoside diphosphate kinase-like	10.07585248	0.0000886602		
XP_001652056.2	Vitellogenic carboxypeptidase			37.57757336	0.00101347
XP_001655104.1	Sorbitol dehydrogenase			27.67324652	0.013266
XP_001663173.2	Solute carrier family 22 member 8			19.16072174	0.000290131
XP_021702456.1	Sodium-coupled monocarboxylate transporter 1 isoform X1			18.47599301	0.000290131
XP_001651077.2	Synaptic vesicle glycoprotein 2B			17.7140231	0.000290131
XP_001656519.2	Solute carrier family 22 member 21 isoform X2			14.01519585	0.000290131
XP_001662495.2	Lipase 1			13.73780875	0.000290131
XP_021706833.1	Sodium-coupled monocarboxylate transporter 1 isoform X2			13.50049126	0.000290131
XP_021698236.1	Solute carrier organic anion transporter family member 2A1			11.93072658	0.000290131
XP_021702682.1	Acidic amino acid decarboxylase GADL1 isoform X3			11.91023521	0.000290131
XP_001649855.2	Sodium/potassium/calcium exchanger 4			11.67450924	0.000290131
XP_021710339.1	Synaptic vesicle glycoprotein 2C			10.26811889	0.000290131
XP_001662720.1	Putative transporter SVOPL			10.08241963	0.000290131
XP_021713275.1	Synaptic vesicle glycoprotein 2A			8.744949179	0.000290131

**FIGURE 3:**
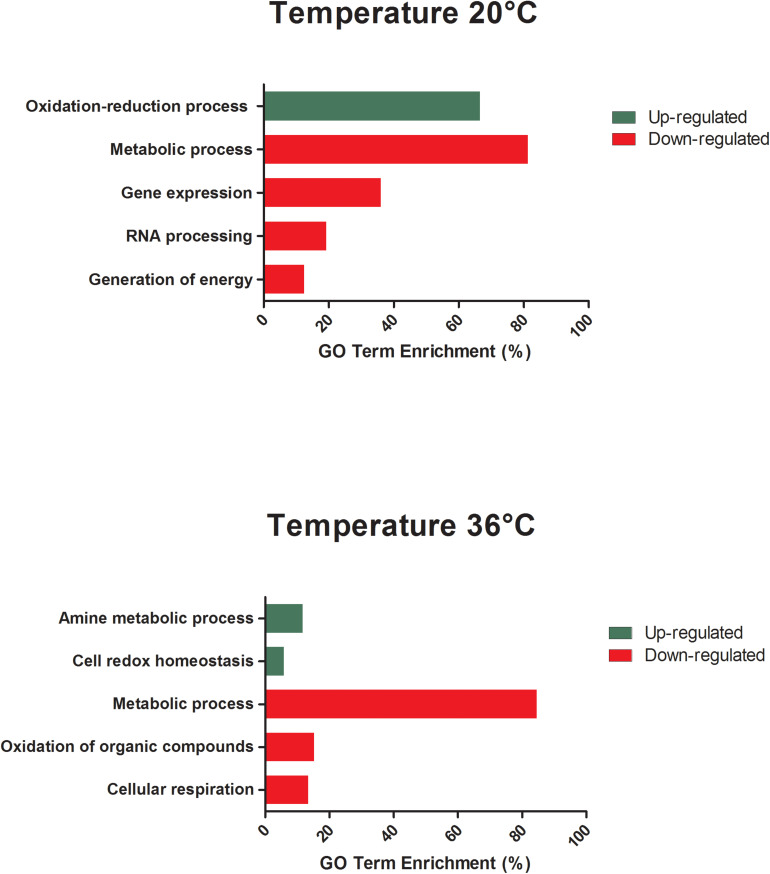
GO term enrichment analysis of differentially expressed genes of unexposed *Aedes aegypti* kept under the lowest (20°C) and the highest (36°C) temperature conditions in the category of biological processes for up- and down-regulated genes at 48 h.

All differentially expressed genes in the midgut at 48 hpf were submitted for gene ontology (GO) analysis to identify cellular processes that were most perturbed in response to temperature treatment, as revealed by the transcriptome profile. GO analysis of the enriched and depleted transcripts from mosquitoes housed at 20°C revealed that 20 enriched GO terms were related to oxidation–reduction processes and 111 depleted GO terms were involved in gene expression, RNA processing, metabolic processes, and generation of energy (Figure 3). Mosquitoes housed at 36°C displayed up-regulated expression related to amine metabolism and cell redox homeostasis processes and down-regulated expression of genes associated with metabolic processes, cellular respiration, and energy derivation by oxidation of organic compounds (Figure 3).

### The Effect of Temperature on Gene Expression in ZIKV-Exposed Mosquitoes

**FIGURE 4:**
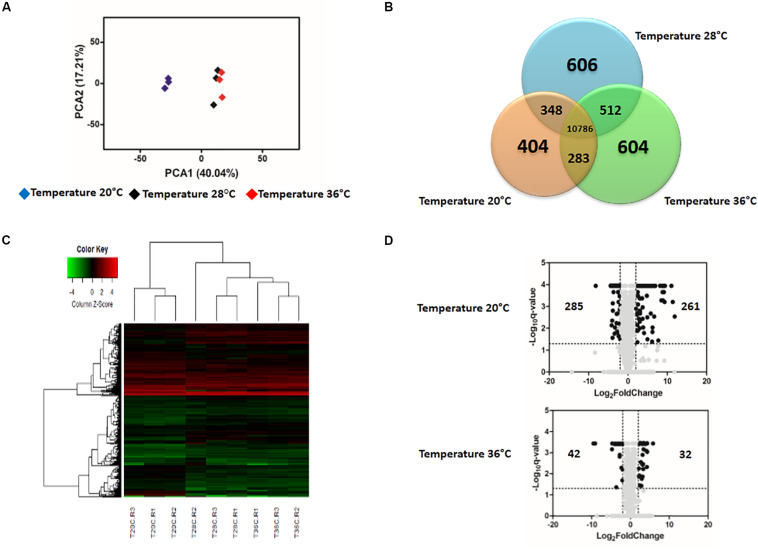
Effect of temperature on the expression profile of Zika-exposed *Aedes aegypti.* **(A)** Principal Component Analysis (PCA) plot showing the global gene expression profiles. **(B)** Venn Diagram reporting the number of specific and shared genes. **(C)** Heatmap plot showing local differences (naming scheme: TC20R1 = replicate 1 for the temperature 20°C). **(D)** Volcano plot representing the differential gene expression in RNAseq samples from infected *Ae. aegypti* exposed to three different constant temperatures (20, 28, and 36°C) at 48 h.

The gene expression profiles in ZIKV-exposed mosquitoes were also significantly affected by environmental temperature and time post-blood feed. In general, the effect of temperature on differential gene expression was similar to patterns observed in non-ZIKV-exposed blood-fed control mosquitoes outlined above. For example, PCA and heatmap analyses on differential gene expression from mosquito midguts at 24 hpf illustrated three distinct groups separated by temperature treatment (Supplementary Figures S2A,C). At 48 hpf, gene expression in mosquitoes housed at 20°C was more distinct than those housed at 28 and 36°C (Figures 4A,C). Further, Venn diagrams demonstrate that 1,416 and 10,786 genes were expressed across all temperatures at 24 hpf (Supplementary Figure S2B) and 48 hpf (Figure 4B), respectively, with the highest overlap in gene expression occurring in mosquitoes housed at 28°C and 36°C at both time points (Supplementary Figure S2B and Figure 4B). Finally, as seen in the absence of ZIKV infection, at 24 hpf, only 1669 and 1797 genes were differentially expressed in mosquitoes housed at 20 and 36°C, respectively, relative to those housed at 28°C. By 48 hpf, we observed a general increase in the number of genes differentially expressed at 20°C (3056, Supplementary Table S3) and a general decrease at 36°C (1518, Supplementary Table S4) relative to mosquitoes housed at 28°C. When we evaluated differential expression through a volcano plot, the profile was similar to that of mosquitoes that were not exposed to ZIKV (Supplementary Figure S2D and Figure 4D).

Several genes that strongly increased at 20°C remained among the top 20 differentially expressed after ZIKV exposure. Lysosomal alpha-mannosidase (XP_021703511.1), two vitellogenins (XP_001660818.2 and XP_001657509.1), phosphoenolpyruvate carboxykinase (XP_001647937.2), protein G12 isoforms (XP_021712126.1, XP_021701760.1, XP_001660827.1, XP_021701761.1, XP_001656377.1, and XP_001656375.1), beta-galactosidase (XP_021705369.1), alpha-N-acetylgalactosaminidase (XP_001650490.2), serine protease SP24D (XP_001659962.1), and chymotrypsin-2 (XP_021698904.1) are some of the genes that remained among the top 20 genes changed by cold temperature in the ZIKV-infection condition. We also saw that although glutamine synthetase (XP_001654186.1) and trypsin (XP_001663002.1) were not listed among the 20 most differentially expressed, they showed enrichment of 50- and 74-fold, respectively. Further, facilitated trehalose transporter Tret1, found to be the most down-regulated in unexposed mosquitoes kept at 36°C, was also the most down-regulated (25-fold) in ZIKV-exposed mosquitoes kept at the same temperature. Finally, serine protease SP24D (XP_001659962.1) and some G12 proteins (XP_021701760.1, XP_021701761.1, XP_001660827.1, and XP_001656377.1) that were enriched at both cool and warm temperatures remained enriched at these temperatures in ZIKV-exposed mosquitoes (Table 2).

**TABLE 2:** Top 20 up- and down-regulated genes of Zika-exposed *Aedes aegypti* in response to low (20°C) and to high (36°C) temperature for 48 h post-blood-feeding relative to standard insectary conditions (28°C).

GENES UP-REGULATED		TEMPERATURE 20°C		TEMPERATURE 36°C	
Gene ID	Gene Description	Fold Change	*q*-value	Fold Change	*q*-value
XP_001657509.1	Vitellogenin-A1-like	3748.76585	0.00288439		
XP_001660818.2	Vitellogenin-A1	2644.35472	0.000624083		
XP_021701760.1	Protein G12	2081.92156	0.000113659	18.9560964	0.00036736
XP_001660827.1	Protein G12	668.331471	0.000113659	9.8533206	0.00036736
XP_021701762.1	Protein G12-like	626.881959	0.000624083	9.79462325	0.00599791
XP_021712126.1	Protein G12-like	580.732514	0.000113659	8.68875819	0.00126501
XP_001663776.1	Protein G12	519.780968	0.000113659	10.3753637	0.000690526
XP_021701761.1	Protein G12	498.942585	0.000113659	9.76777521	0.00036736
XP_021698904.1	Chymotrypsin-2	476.004037	0.000113659		
XP_001657506.2	Vitellogenin-A1-like	467.414928	0.000526591		
XP_001656377.1	Protein G12 isoform X2	400.561513	0.000113659	8.94238711	0.00036736
XP_021702099.1	Probable nuclear hormone receptor HR3 isoform X5	356.184271	0.000526591		
XP_001656375.1	Protein G12	315.925373	0.000113659		
XP_001659962.1	Serine protease SP24D	315.50958	0.000113659	8.63508273	0.00036736
XP_021705369.1	Beta-galactosidase	310.614205	0.000113659		
XP_001650490.2	Alpha-N-acetylgalactosaminidase	292.584595	0.000113659		
XP_001652055.1	Vitellogenic carboxypeptidase-like	206.875645	0.0359751		
XP_001652056.2	Vitellogenic carboxypeptidase	123.386436	0.000113659		
XP_001647937.2	Phosphoenolpyruvate carboxykinase [GTP]	106.495599	0.000113659		
XP_021703511.1	Lysosomal alpha-mannosidase isoform X2	103.072096	0.000113659		
XP_001652358.2	Peritrophin-1			55.8211523	0.00036736
XP_021697715.1	Peritrophin-1-like			16.3831402	0.00036736
XP_001658471.2	Mite group 2 allergen Gly d 2.01			15.940003	0.00036736
XP_001663895.2	Trypsin 5G1			9.16859073	0.00365688
XP_001660673.2	Trypsin 5G1-like			8.69411995	0.0136618
XP_001661186.2	Protein G12			7.2863687	0.00036736
XP_001663102.2	Malate synthase			6.86576273	0.00036736
XP_001663439.2	Collagenase			6.39130263	0.00279659
XP_021707618.1	Probable chitinase 2			6.27684681	0.00036736
XP_001651623.2	Surface antigen CRP170			5.38042449	0.00036736
XP_001653091.2	Trypsin alpha-3			5.26940475	0.00036736
XP_001659961.1	Chymotrypsin-2			5.00475692	0.00036736

**GENES DOWN-REGULATED**		**TEMPERATURE 20°C**		**TEMPERATURE 36°C**	
	**Gene Description**	**Fold Change**	***q*-value**	**Fold Change**	***q*-value**

XP_001663064.2	UDP-glucuronosyltransferase 2B18	22.0442425	0.000113659	5.62311675	0.00036736
XP_011493503.2	H/ACA ribonucleoprotein complex subunit 1	20.5197322	0.000113659		
XP_001654398.2	rRNA 2’-O-methyltransferase fibrillarin	19.1980783	0.000113659		
XP_001659197.1	DNA-directed RNA polymerase III subunit RPC10	18.0109204	0.00732014		
XP_001658147.1	Facilitated trehalose transporter Tret1	17.354405	0.000113659	25.7984056	0.00036736
XP_001663005.2	Periodic tryptophan protein 1 homolog	16.363733	0.000113659		
XP_021698953.1	CAD protein	16.0097625	0.00443189		
XP_001655305.2	Protein MAK16 homolog A	15.3024421	0.000113659		
XP_001656228.2	COX assembly mitochondrial protein 2 homolog	14.7057657	0.000721477		
XP_021706766.1	Glycerol-3-phosphate dehydrogenase mitochondrial isoform X1	14.6844772	0.000113659		
XP_001649209.1	RRP15-like protein isoform X2	14.5214136	0.00156177		
XP_001652303.1	mRNA turnover protein 4 homolog	14.3570755	0.000113659		
XP_001662723.2	Titin homolog	14.3017521	0.000113659		
XP_021699084.1	Proton-coupled amino acid transporter 1-like	14.2666035	0.000113659		
XP_001658562.1	Activator of basal transcription 1	13.971742	0.000113659		
XP_021695012.1	46 kDa FK506-binding nuclear protein	13.8346093	0.000113659		
XP_001660583.2	Glutamate-rich WD repeat-containing protein 1	13.2594589	0.000113659		
XP_021703839.1	Protein Notchless	13.0245353	0.000113659		
XP_001656680.1	NHP2-like protein 1 homolog	12.0716418	0.000113659		
XP_001655996.2	H/ACA ribonucleoprotein complex non-core subunit NAF1	11.9572191	0.000113659		
XP_001662495.2	Lipase 1			16.3418567	0.00036736
XP_021698905.1	Chymotrypsin-2			13.9310298	0.00036736
XP_001649987.2	Synaptic vesicle glycoprotein 2B			12.6667105	0.0434966
XP_021702682.1	Acidic amino acid decarboxylase GADL1 isoform X3			11.6866538	0.00036736
XP_001649098.2	Probable cytochrome P450 9f2			8.68297843	0.00036736
XP_021699787.1	Solute carrier family 2 facilitated glucose transporter member 3			7.85777033	0.00036736
XP_001652075.2	Venom protease			6.9627634	0.00153067
XP_021702456.1	Sodium-coupled monocarboxylate transporter 1 isoform X1			6.31621377	0.00036736
XP_001651954.2	Trypsin			6.12067175	0.00036736
XP_001649855.2	Sodium/potassium/calcium exchanger 4			5.8573577	0.00036736
XP_021704942.1	Excitatory amino acid transporter 1			5.56974007	0.00036736
XP_021708608.1	Putative helicase MOV-10			5.4055447	0.00036736
XP_001656046.1	Alanine–glyoxylate aminotransferase 2-like			5.35883724	0.00036736
XP_021698609.1	Chymotrypsin-2			5.27740974	0.00036736
XP_001661015.2	Putative alpha-L-fucosidase			5.02108797	0.00036736
XP_001658000.3	Glutathione S-transferase 1			5.0085396	0.00517339
XP_021704288.1	Phosphotriesterase-related protein			4.96319797	0.00036736
XP_001661388.1	Chymotrypsin-1			4.7758913	0.00036736

Despite these similarities, we did note some key differences between the top 20 genes most differentially expressed in unexposed (Table 1) and ZIKV-exposed mosquitoes (Table 2), with the greatest change in expression reflected in mosquitoes housed at 20°C and 36°C at 48 h relative to those housed at 28°C. In unexposed mosquitoes kept at 20°C, vitellogenin-A1-like (XP_001657509.1) and vitellogenin-A1 (XP_001660818.2) were up-regulated, yet ZIKV exposure amplified the enrichment from 138- and 68-fold to 3748- and 2644-fold (Tables 1, 2). Surprisingly, in ZIKV-exposed mosquitoes, we did not detect a large up-regulation of Hsp70 at 36°C like we did in the unexposed population.

**FIGURE 5:**
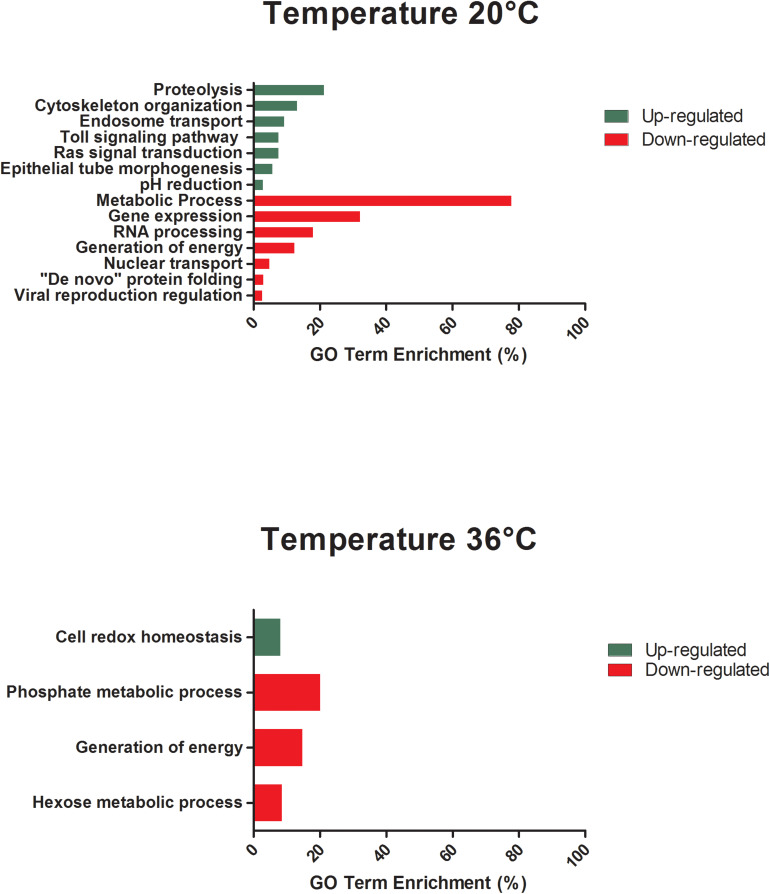
GO term enrichment analysis of differentially expressed genes of *Aedes aegypti* exposed to Zika virus under the lowest (20°C) and in the highest (36°C) temperature conditions in the category of biological processes for up- and down-regulated genes at 48 h.

The GO analysis of differentially expressed genes at 48 hpf in ZIKV-exposed mosquitoes demonstrated distinct effects of cool and warm temperatures on cellular and metabolic function relative to mosquitoes housed at 28°C (Figure 5). Further, the functions of these differentially expressed genes were, in part, different from those in unexposed mosquitoes housed at these temperatures (Figure 3). For example, when maintained at 20°C, oxidative-reduction processes were no longer enriched, as was seen in unexposed mosquitoes. Instead, ZIKV-exposed mosquitoes housed at 20°C had significant enrichment of the Toll signaling pathway, a known anti-dengue pathway in *Ae. aegypti* (Tchankouo-Nguetcheu et al., 2010). Additionally, endosome transport, Ras protein signal transduction, actin cytoskeleton organization, epithelial tube morphogenesis, pH reduction, and proteolysis were enriched. In contrast, nuclear transport, regulation of viral reproduction, “*de novo*” protein folding, generation of energy, gene expression, RNA processing, and metabolic processes were down-regulated in addition to the expression associated with gene expression, RNA processing, and metabolic processes observed in unexposed counterparts at this temperature (Figure 5). ZIKV-exposed mosquitoes housed at 36°C no longer had significant enrichment in genes associated with cellular amine processes and had significant depletion in genes associated with hexose and phosphate metabolic processes and with the generation of precursor metabolites and energy (Figure 5).

### Effects of Temperature on Oxidative Stress and Innate Immune Mechanisms

To further explore the effects of temperature on uninfected and ZIKV-exposed mosquitoes, we highlight differences in those genes involved in managing oxidative stress, innate immunity, and apoptosis at both 20 and 36°C compared to 28°C at 48 hpf (Supplementary Tables S5, S6 and Supplementary Figures S3, S4). Virus infection has previously been shown to be modified by complex responses related to detoxification of the blood meal, metabolism, immunity, and apoptosis in some systems (Sanders et al., 2005; Girard et al., 2010; Tchankouo-Nguetcheu et al., 2010; Colpitts et al., 2011; Wang et al., 2012; Neill et al., 2015; Eng et al., 2016). Further, these responses need not respond equivalently to temperature variation, as shown in previous work, which demonstrated that mosquito immune responses differed qualitatively and quantitatively across a range of environmental temperatures (Murdock et al., 2012b). The majority of genes involved in managing oxidative stress, innate immunity, and apoptosis exhibited qualitatively different patterns in gene expression in response to cool and warm temperatures in uninfected and ZIKV-exposed mosquitoes. However, while significant, the majority of these differences were very subtle (<2.0-fold; Supplementary Figures S3, S4). We did observe components of the melanization and Toll pathways to be modestly expressed (>2.0 fold; Supplementary Figures S3, S4) in response to temperature. An isoform of phenoloxidase (XP_021699380.1), a c-type lectin (XP_001661643.1), and the Toll receptor 6 (TLR6) (XP_021712805.1) were significantly enriched in both uninfected and ZIKV-exposed mosquitoes housed at 20°C.

### Mosquito Responses to ZIKV Infection Are Remarkably Modulated by Environmental Temperature

**FIGURE 6:**
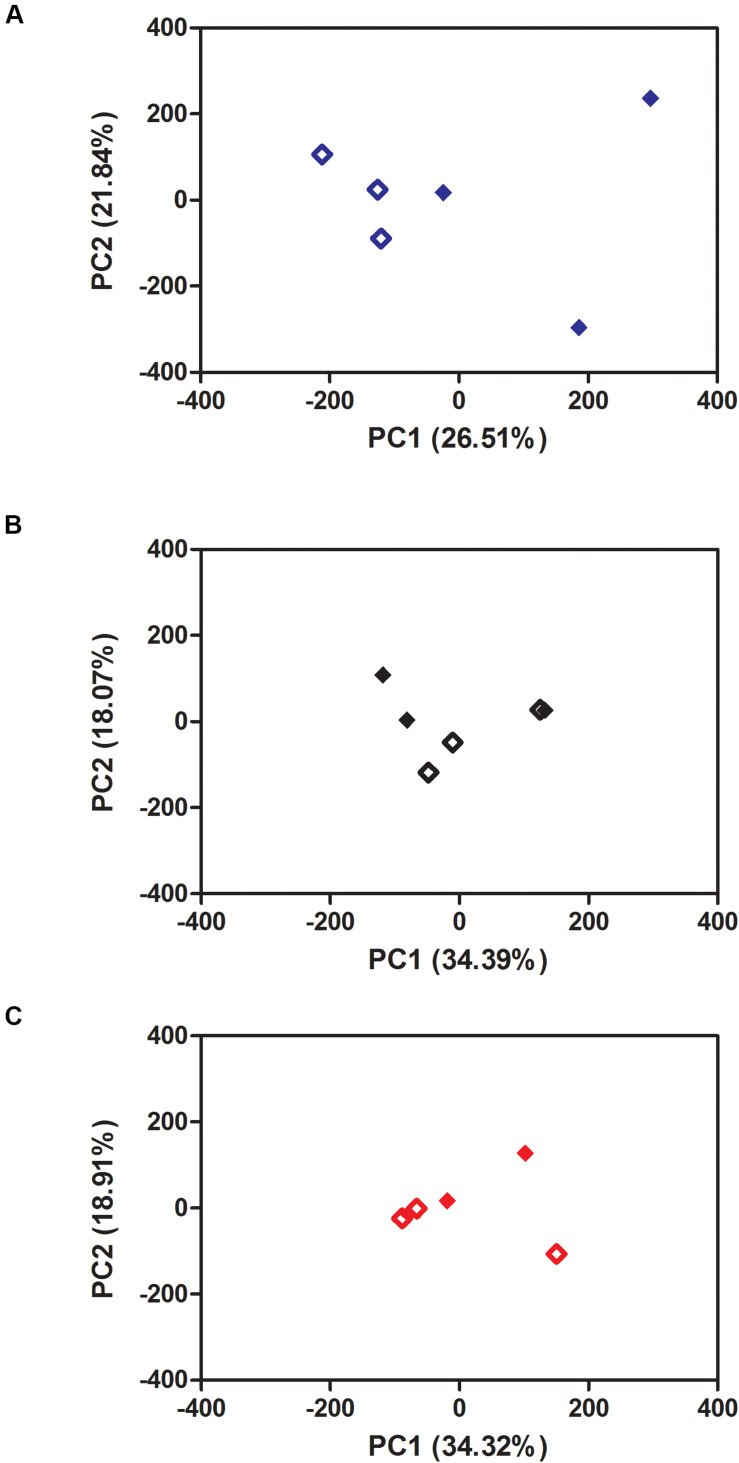
Principal Component Analysis (PCA) of the effect of Zika exposure on *Aedes aegypti* gene expression at three different constant temperatures: **(A)** 20°C, **(B)** 28°C, **(C)** 36°C at 48 h. Open diamond: non-exposed mosquitoes. Closed diamond: Zika-exposed mosquitoes.

**FIGURE 7:**
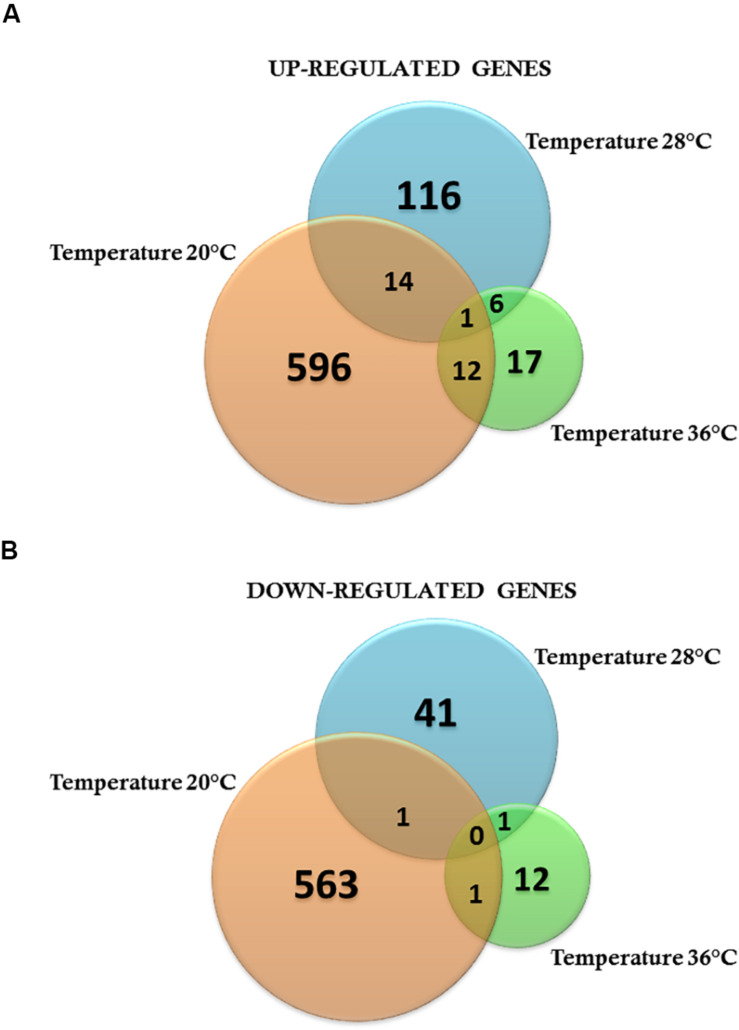
Venn diagram representing the number of specific differentially expressed genes for Zika-infected *Aedes aegypti* exposed to three different constant temperatures (20, 28, and 36°C). **(A)** Up-regulated genes. **(B)** Down-regulated genes at 48 h.

To investigate the effects of ZIKV-exposure on global gene-expression patterns from mosquito midguts early on in the infection process, we compared gene expression between ZIKV-exposed and unexposed mosquitoes within each temperature treatment. The PCA plots demonstrate that ZIKV exposure does not alter mosquito transcription at 24 hpf (Supplementary Figure S5) within a given temperature treatment. However, 48-hpf ZIKV-exposed and unexposed samples under the cold temperature condition were distributed in two distinct groups, although one of the ZIKV-exposed biological replicates was relatively close to the control group (Figure 6A). Also, the overall number of differentially expressed genes between ZIKV-exposed and unexposed mosquitoes varied across temperature treatments. For example, at 24 hpf, we observed a total of 225, 154, and 161 genes differentially expressed between ZIKV-exposed and unexposed mosquitoes at 20°C, 28°C, and 36°C, respectively (Supplementary Tables S7–S9). We identified only two proteins – a sodium/potassium/calcium exchanger 4 (XP_001649855.2) and an uncharacterized protein (XP_001654261.2) – that were up-regulated in ZIKV-exposed mosquitoes at all temperatures (Supplementary Figure S6A) and one protein – chymotrypsin-2 (XP_021698609.1) that was down-regulated (Supplementary Figure S6B). At 48 hpf, we observed more genes to be differentially expressed (1188 genes) in mosquitoes housed at 20°C between ZIKV-exposed and unexposed mosquitoes, versus only 180 and 50 genes at 28 and 36°C, respectively (Supplementary Tables S7–S9). Only one uncharacterized protein (XP_001658660.2) was up-regulated in the ZIKV-exposed mosquitoes at all temperatures (Figures 7A,B) at this sampling time point. These results indicate that while the physiological responses of mosquito midguts to ZIKV exposure early in the infection process are similar within a given temperature treatment, these responses are significantly distinct across different environmental temperatures.

When concentrating on the top 10 most differentially expressed genes between ZIKV-exposed and unexposed mosquitoes at each temperature treatment at 48 hpf (Table 3), only at 20°C did we observe genes with altered expression (enrichment or depletion) of 10-fold or more. GO analysis demonstrated that functions associated with these genes included metallopeptidases (angiotensin-converting enzyme and vitellogenic carboxypeptidase), ammonia/nitrogen metabolism (argininosuccinate lyase and alanine aminotransferase 1), and iron ion binding (a member of the cytochrome P450 family, 4g15).

**TABLE 3:** Top 10 up- and down-regulated genes of Zika-exposed *Aedes aegypti* relative to uninfected mosquitoes kept at low (20°C), standard (28°C), and high (36°C) temperatures 48 h post-blood-feeding.

GENES UP-REGULATED		TEMPERATURE 20°C		TEMPERATURE 28°C		TEMPERATURE 36°C	
Gene ID	Gene Description	Fold Change	*q*-value	Fold Change	*q*-value	Fold Change	*q*-value
XP_011493087.1	Angiotensin-converting enzyme	14.1809276	0.000429978				
XP_001652055.1	Vitellogenic carboxypeptidase-like	13.75772483	0.000429978				
XP_001657506.2	Vitellogenin-A1-like	11.96534425	0.000429978				
XP_001657509.1	Vitellogenin-A1-like	11.70010847	0.000429978				
XP_001660818.2	Vitellogenin-A1	10.9320344	0.000429978				
XP_001652056.2	Vitellogenic carboxypeptidase	10.74370729	0.000429978				
XP_001660472.2	Alanine aminotransferase 1	10.39479931	0.000429978				
XP_001656695.1	Argininosuccinate lyase	10.17572724	0.000429978				
XP_001648376.1	Cytochrome P450 4g15	10.00001321	0.000429978				
XP_001659164.1	Leucine-rich repeat transmembrane neuronal protein 3	9.612555348	0.000429978				
XP_001649098.2	Probable cytochrome P450 9f2			4.118816919	0.0027631		
XP_001659492.2	Serine protease SP24D			3.289321443	0.0027631		
XP_021698905.1	Chymotrypsin-2			3.165229262	0.0027631		
XP_001661721.2	Solute carrier family 45 member 4			3.060526797	0.0027631		
XP_001654886.2	Zinc carboxypeptidase A 1			2.871948741	0.0027631		
XP_001661388.1	Chymotrypsin-1			2.816727502	0.0027631		
XP_021698904.1	Chymotrypsin-2			2.669611858	0.0027631		
XP_021707253.1	Lipase member H			2.612389473	0.0027631		
XP_021697282.1	Multiple inositol polyphosphate phosphatase 1			2.446636883	0.0027631		
XP_001658491.2	Trypsin 5G1-like			2.279646301	0.0027631		
XP_001652358.2	Peritrophin-1					8.437408782	0.00630758
XP_001648381.1	UNC93-like protein					7.430915686	0.00630758
XP_021710339.1	Synaptic vesicle glycoprotein 2C					5.329166839	0.00630758
XP_021697715.1	Peritrophin-1-like					4.823164447	0.00630758
XP_011493129.2	Flocculation protein FLO11					2.282808753	0.00630758
XP_021706761.1	Cysteine sulfinic acid decarboxylase					2.236697262	0.00630758
XP_021707618.1	Probable chitinase 2					2.214805485	0.00630758
XP_001658086.2	Peptidoglycan recognition protein 1					2.009494011	0.00630758
XP_001649855.2	Sodium/potassium/calcium exchanger 4					1.947834362	0.00630758
XP_021709756.1	ATP-binding cassette sub-family A member 3 isoform X1					1.930421649	0.00630758
XP_001659797.2	Beta-1.3-glucan-binding protein	8.48073909	0.000429978				
XP_001657206.1	Cytochrome P450 9e2	4.355565374	0.000429978				
XP_001652358.2	Peritrophin-1	2.847448995	0.000429978				
XP_021699084.1	Proton-coupled amino acid transporter 1-like	2.551861447	0.000429978				
XP_001659796.1	Beta-1.3-glucan-binding protein	2.517270058	0.000429978				
XP_001649098.2	Probable cytochrome P450 9f2	2.415932254	0.000429978				
XP_001649745.1	Very long-chain specific acyl-CoA dehydrogenase mitochondrial	2.281021429	0.000429978				
XP_001654398.2	rRNA 2’-O-methyltransferase fibrillarin	2.204742243	0.000429978				
XP_001661250.1	Peroxiredoxin-6	1.971170346	0.000429978				
XP_001649797.1	Peptide methionine sulfoxide reductase	1.965683434	0.000429978				
XP_001652056.2	Vitellogenic carboxypeptidase			6.420385839	0.0027631		
XP_001655729.2	Tryptase			5.300762859	0.0027631		
XP_001647719.2	Transferrin			4.298907297	0.0027631		
XP_001655031.2	Carbonic anhydrase 2			3.707662693	0.0027631		
XP_011493147.1	Glycine-rich protein 5			3.626857343	0.0027631		
XP_001651077.2	Synaptic vesicle glycoprotein 2B			3.02148965	0.0027631		
XP_001659383.2	Angiopoietin-related protein 1			2.46044625	0.0027631		
XP_001651411.2	Lysosomal alpha-mannosidase			2.427598779	0.0027631		
XP_001656519.2	Solute carrier family 22 member 21 isoform X2			2.425681278	0.0027631		
XP_011493149.1	Hyphally-regulated protein			2.384835903	0.0049383		
XP_021696806.1	Pupal cuticle protein Edg-78E					18.1975207	0.00630758
XP_011493274.2	Extensin					3.699473591	0.00630758
XP_001661011.1	Protein lethal(2)essential for life					3.215359388	0.00630758
XP_001658359.2	Brachyurin					2.357013464	0.00630758
XP_001649783.1	Maltase 1					1.680632876	0.0148679
XP_001647586.2	Asparagine synthetase [glutamine-hydrolyzing] 1					1.637484216	0.00630758
XP_021694990.1	Myrosinase 1-like					1.626761779	0.00630758
XP_021693649.1	Heat shock protein 70 A1					1.55902061	0.02715
XP_001651895.1	Acyl-CoA:lysophosphatidylglycerol acyltransferase 1					1.523434001	0.00630758
XP_001649752.1	Heat shock protein 83					1.48484568	0.00630758

Interestingly, two vitellogenins (XP_001660818.2 and XP_001657509.1) that were strongly up-regulated in ZIKV-exposed and unexposed mosquitoes housed at 20°C relative to those housed at 28°C were the genes most enriched by ZIKV exposure at the cold temperature (Table 3). The expression of these genes did not change in response to ZIKV exposure at 28°C (Supplementary Table S8) and 36°C (Supplementary Table S9), suggesting that cold stress alters the midgut vitellogenin expression and may be more significant during a viral infection. ZIKV exposure induced a depletion of beta-1,3-glucan-binding protein (GNBP), which binds to β−1,3-glucan and lipopolysaccharide on the surface of pathogens (Dimopoulos et al., 1997), when mosquitoes were maintained at 20°C. Finally, among the most down-regulated genes in ZIKV-exposed mosquitoes at 28°C, solute carrier family 22 (XP_001656519.2), synaptic vesicle glycoprotein (XP_001651077.2), and vitellogenic carboxypeptidase (XP_001652056.2) (Table 3) were also among the most down-regulated genes in unexposed mosquitoes housed at 36°C relative to 28°C (Table 1).

## Discussion

The dynamics and distribution of vector-borne diseases depend on the interplay between the pathogen, the mosquito, and the environment. Temperature is a strong driver of vector-borne disease transmission (Kilpatrick et al., 2008; Lambrechts et al., 2011; Carrington et al., 2013a, b; Mordecai et al., 2013, 2017; Johnson et al., 2015; Shocket et al., 2018; Tesla et al., 2018a). Despite the strong effects of temperature on mosquito-borne pathogens, little is known about the underlying mechanisms involved (Adelman et al., 2013). In this study, RNA sequencing of *Ae. aegypti* midguts unexposed or exposed to ZIKV, taken early in the infection process, revealed different transcriptional responses to variation in environmental temperature, with ZIKV infection modifying these responses to temperature.

Previously, we found that temperature significantly affected the efficiency of the establishment of ZIKV infection in *Ae. aegypti* midguts, with cool temperatures limiting ZIKV transmission primarily due to poor midgut infection, slow replication, and poor dissemination, while high mosquito mortality at warmer temperatures inhibited ZIKV transmission despite efficient ZIKV infection (Tesla et al., 2018a). Similar to our previous study, we were unable to detect ZIKV replication when mosquitoes were housed in cool conditions (Figure 1), which is similar to findings for other flavivirus systems in which infection rates were measured across different constant temperatures (Watts et al., 1987; Kilpatrick et al., 2008; Johansson et al., 2012; Xiao et al., 2014). There was no significant difference in the viral RNA levels quantified from the three temperatures at 24 hpf, reflecting the initial concentration of viral RNA ingested in the blood meal (Figure 1). Therefore, we can confirm that while all treatment groups obtained ZIKV in the blood meal, only the mosquitoes housed at standard (28°C) or warm (36°C) temperatures were actively replicating virus by 48 hpf.

We found that variation in temperature elicited strong expression responses in unexposed and ZIKV-exposed mosquitoes. We observed that mosquitoes housed at a cold temperature (20°C) had more genes differentially expressed at 48 hpf relative to mosquitoes housed in standard (28°C) and warm environments (36°C), which exhibited more similar patterns in gene expression (Figures 2A, 4A). This is not entirely surprising, as metabolic theory predicts that low body temperatures will inevitably depress the rates of biochemical reactions (Angilletta et al., 2010). Our data support this hypothesis, as many of the genes drastically altered at 20°C participate in blood meal digestion, peritrophic membrane (PM) formation, metabolism, and managing oxidative stress associated with the breakdown of hemoglobin into heme, a cytotoxic product (Tables 1, 2). Phosphoenolpyruvate carboxykinase and trypsin are upregulated in *Ae. aegypti* midgut during the first few hours after ingestion of a blood meal (Sanders et al., 2003) but were extraordinarily up-regulated in mosquitoes 48 hpf when housed in a cool environment in our study. Additionally, protein G12, which has previously been associated with blood meal digestion and nitrile-specific detoxification (Morlais et al., 2003; Fischer et al., 2008; Bonizzoni et al., 2011), was one of the most enriched transcripts. Further, two digestive proteases involved in glycoside hydrolysis, beta-galactosidase and alpha-N-acetylgalactosaminidase (Santamaría et al., 2015), were highly induced at 48 hpf. Glutamine synthetase, an enzyme that contributes to PM formation, was also highly induced, further demonstrating that cool temperatures delay blood meal digestion (Supplementary Tables S1, S3). Although the PM is semi-permeable, it is thought to form a barrier and protect the midgut from pathogens [e.g., viruses (Wang and Granados, 2000), bacteria (Kuraishi et al., 2011; Jin et al., 2019), malaria (Rodgers et al., 2017), and protozoa (Weiss et al., 2014)] and other harmful substances present in the insect gut after a blood meal (Wang et al., 2004; Shibata et al., 2015).

We also observed several genes involved in the innate immune response to be modestly upregulated in response to 20°C relative to their levels under warmer temperature conditions in both unexposed and ZIKV-exposed mosquitoes. Melanization is a major effector mechanism of the mosquito immune response and has been implicated in the defense against a diversity of pathogens [e.g., bacteria (Hillyer et al., 2003a, b), malaria (Kumar et al., 2003; Jaramillo-Gutierrez et al., 2009), filarial worms (Christensen et al., 2005; Huang et al., 2005), and viruses (Rodriguez-Andres et al., 2012)]. Phenoloxidase, a key enzyme in the melanization pathway, was up-regulated in mosquito midguts at 20°C (Supplementary Figures S3B, S4B). Studies in both butterflies and *Anopheles stephensi* demonstrated that phenoloxidase activity was higher at cool temperatures and becomes less efficient at warmer temperatures (Suwanchaichinda and Paskewitz, 1998; Murdock et al., 2012b). The production of melanin is also essential for other physiological processes such as cold acclimatization in insects (Crill et al., 1996; Kutch et al., 2014), the formation of the hard protective layer around eggs, and wound healing (Lai et al., 2009). Our data also reveal that c-type lectin, reported to participate in the activation of the melanization cascade (Yu and Kanost, 2000; Christensen et al., 2005), was also up-regulated. Therefore, our results suggest that cold stress triggers numerous molecular changes in the mosquito, including modest changes in the levels of important immune effectors that could have important consequences for arboviral infection.

Contrary to what we observed at the cool temperature, exposure to hot conditions (36°C) does not trigger pronounced up- or down-regulation of genes relative to standard conditions (28°C). The heat shock protein 70 (HSP 70) transcript was most enriched in response to the hot environment (Table 1). The upregulation of HSP 70 is associated with reduced lifespans in other insect systems (Feder and Krebs, 1998; Feder, 1999), which may explain the rapid mosquito mortality we observed at this temperature in previous work (Tesla et al., 2018a). HSP70 has also been suggested to facilitate arbovirus infection in mosquitoes in terms of viral entry, viral RNA synthesis, and virion production (Kuadkitkan et al., 2010; Taguwa et al., 2015).

Zika virus exposure induced very modest effects when comparing ZIKV-exposed and unexposed mosquitoes reared at 28 and 36°C, while those that experienced the cool temperature exhibited a larger alteration in gene expression at 48 hpf (Figure 6 and Supplementary Figure S5). This may not be entirely surprising, as ZIKV-induced transcriptional changes under standard rearing conditions (28°C) have previously been shown to be subtle 48 h post-infection (Murdock et al., 2012a) and ZIKV was only observed to be actively replicating at 28 and 36°C. When concentrating on differentially expressed genes between ZIKV-exposed and unexposed mosquitoes at each temperature treatment (Table 3), only at 20°C do we observe changes of 10-fold or more. Therefore, the presence of ZIKV in the blood meal did alter the response of mosquitoes to temperature variation, with the most pronounced differences occurring in mosquitoes housed at the cool temperature. In particular, the midguts of ZIKV-exposed mosquitoes experienced enhanced signal transduction processes, pH modification, midgut epithelial morphogenesis, and Toll pathway activation relative to ZIKV-exposed mosquitoes at 28°C (Figure 5), and this pattern was qualitatively different to a similar comparison in unexposed mosquitoes (Figure 3). These changes could be reflective of patterns observed in other studies demonstrating that mosquitoes infected with blood-borne pathogens actively modify ROS metabolism in midgut cells to control levels of hydrogen peroxide (H_2_O_2_), which in turn is an important modulator of downstream innate immune responses (e.g., Toll pathway), antimicrobial peptide production, and pathogen infection (Molina-Cruz et al., 2008; Herrera-Ortiz et al., 2011; Oliveira et al., 2011, 2012). Furthermore, the presence and abundance of particular microbial flora [e.g., *Wolbachia* (Pan et al., 2012)] and the proliferation of the midgut flora due to ingestion of the blood meal (Carissimo et al., 2014; Saraiva et al., 2016; Barletta et al., 2017) can also trigger ROS production, with temperature variation modifying these effects.

Additionally, vitellogenin proteins (Vg) were highly upregulated (>3000 fold) when ZIKV-exposed mosquitoes were housed at cool temperatures relative to ZIKV-exposed mosquitoes at 28°C (Table 2) and unexposed mosquitoes at 20°C (Table 3). Vg is a precursor egg-yolk protein but may also function by shielding cells from the negative effects of inflammation and infection (Corona et al., 2007; Azevedo et al., 2011). Work with honey bees suggests that Vg-incubated insect cells display enhanced tolerance against H_2_O_2_-induced oxidative stress (Fluri et al., 1977; Seehuus et al., 2006). Vg also binds to dying cells, suggesting that it may play a role in recognizing damaged cells and shielding healthy cells from toxic by-products (Havukainen et al., 2013). Both caspase dronc [an inhibitor caspase (Cooper et al., 2007)] and an effector caspase (Bryant et al., 2008), which are important components of the apoptotic pathway controlling mechanisms of cell death, were enriched in mosquitoes housed at 20°C (Supplementary Figures S3E, S4E). Thus, elevated Vg expression may combat cellular damage resulting from elevated heme toxicity and oxidative stress (Havukainen et al., 2013) associated with the delayed breakdown of hemoglobin in the blood meal (Bottino-Rojas et al., 2015) in mosquitoes housed at cool temperatures. There may also be a direct effect of Vg on ZIKV, as it has been associated with antiviral effects in some fish species (Garcia et al., 2010). Entry of ZIKV into mammalian cells is associated with apoptotic mimicry, with viral lipids interacting with phosphatidylserine receptors on cells gaining entry in a pathway similar to clearance of apoptotic cells (Hamel et al., 2015). Honey bee Vg binds to dying cells through interactions with lipids (Havukainen et al., 2013). Although the receptors ZIKV uses to interact and enter mosquito cells have not been identified, if Vg protein coats viral particles, it may impede normal cellular interaction and entry. Alternatively, ZIKV infection could be limited at the cooler temperature if infected mosquitoes balance ROS metabolism toward a higher state of oxidative stress, as shown in other systems (Molina-Cruz et al., 2008; Herrera-Ortiz et al., 2011; Pan et al., 2012; Bottino-Rojas et al., 2015; Wong et al., 2015), facilitating downstream innate immune mechanisms and virus killing. Whether overexpressions of Vg lipoproteins have direct effects on ZIKV infection or reflect a response to buffer ZIKV-exposed mosquitoes to a higher state of oxidative stress in the midgut remain open questions that will be explored in future experiments.

Finally, in ZIKV-exposed mosquitoes at 36°C, we observed peritrophin, one of the components of the peritrophic matrix, to be highly up-regulated relative to ZIKV-exposed mosquitoes housed at 28°C (Table 2) and unexposed mosquitoes housed at 36°C (Table 3). Although PM formation is highly induced 3–24 hpf, peritrophin can undergo positive modulation by pathogens in other vector-borne disease systems (e.g., *Le. major*) (Coutinho-Abreu et al., 2013). While we cannot confirm whether HSP70 or modulation of peritrophin play a role in ZIKV infection, these could be potential mechanisms explaining why we detect higher viral RNA levels at 36°C in this study (Figure 1) and efficient viral dissemination and salivary gland invasion in previous work (Tesla et al., 2018a).

Although we estimated the effects of mean constant temperatures on the immune-physiological profiles of *Ae. aegypti* in response to ZIKV infection to maintain experimental tractability, mosquitoes and their pathogens live in a variable world where temperatures fluctuate daily and seasonally (easily encompassing the range of temperatures explored here). Both mosquito immunity and virus transmission have previously been shown to differ in fluctuating environments relative to constant temperature environments (Lambrechts et al., 2011; Carrington et al., 2013a, b; Murdock et al., 2013). There is also a substantial body of work demonstrating carry-over effects of environmental variation in the larval environment on adult mosquito phenotypes, fitness, and metrics of transmission (Muturi and Alto, 2011; Muturi et al., 2011a, b; Alto and Bettinardi, 2013; Buckner et al., 2016; Evans et al., 2018). While outside the scope of this study, future work should investigate how both fluctuating environmental conditions and the larval environment modify the physiological and immunological responses of adult mosquitoes to arbovirus infection and temperature variation in the adult environment. Finally, while there is currently limited evidence demonstrating that mosquitoes select microhabitats to optimize internal body temperatures for metabolic demands (Blanford et al., 2009), as in other ectothermic organisms (Huey, 1991; Hertz et al., 1994; Gvoždík, 2002; Dillon et al., 2009), if this did occur in the field, it would modify the range of field-relevant temperatures mosquitoes effectively experience.

In this study, we demonstrate profound effects of temperature on ZIKV viral replication and the transcriptional responses of mosquitoes. Temperature variation may alter the ZIKV infection process either through modifying the response of mosquitoes to ZIKV infection, altering the efficiencies of viral-specific processes, or, more likely, both. Our study focused on midgut responses early in the infection process. However, disentangling these effects will require the sampling of other immunological tissues and at later time points where high levels of ZIKV RNA can be detected. While further work is needed to determine the precise mechanisms at play, our results indicate that temperature shifts the balance and dynamics of the midgut environment, which could result in direct and indirect consequences for the ZIKV-infection process. These results do reinforce the assertion that the conventional approach of studying the mechanisms underpinning mosquito–pathogen interactions under a narrow set of laboratory conditions or across canonical innate immune pathways is likely missing important biological complexity. To move forward, we need to begin framing our mechanistic understanding of this dynamic phenotype in the ecologically variable world in which mosquitoes and pathogens associate. This study represents a key advance toward this objective.

## Data Availability Statement

The fast raw data were deposited in the NCBI SRA database under accession number PRJNA615972. This Sequence Read Archive (SRA) submission will be released on 2020–10–20 or upon publication, whichever is first.

## Ethics Statement

All mosquito infection work with ZIKV was approved by the University of Georgia, Athens Institutional Biosafety Committee (reference number 2015-0039).

## Author Contributions

BT performed the experiments. PF and TO analyzed the RNAseq data. CM, MB, TO, and LN designed the experiments, acquired the funding, and supervised the project. All authors wrote the manuscript.

## Conflict of Interest

The authors declare that the research was conducted in the absence of any commercial or financial relationships that could be construed as a potential conflict of interest.

## Footnotes

1 https://www.graphpad.com/quickcalcs/Grubbs1.cfm

2 https://www.ncbi.nlm.nih.gov/

3 www.graphpad.com

4 https://www.ncbi.nlm.nih.gov/sites/batchentrez
