# Supplementary material for: Temperature Dramatically Shapes Mosquito Gene Expression With Consequences for Mosquito–Zika Virus Interactions
Source: Front Microbiol. 2020 Jun 12;11:901. doi: 10.3389/fmicb.2020.00901 (PMC7303344; doi:10.3389/fmicb.2020.00901)
Supplement: Supplementary file 1 [file Data_Sheet_1.pdf]

Supplementary Material and Captions

Supplementary Figures

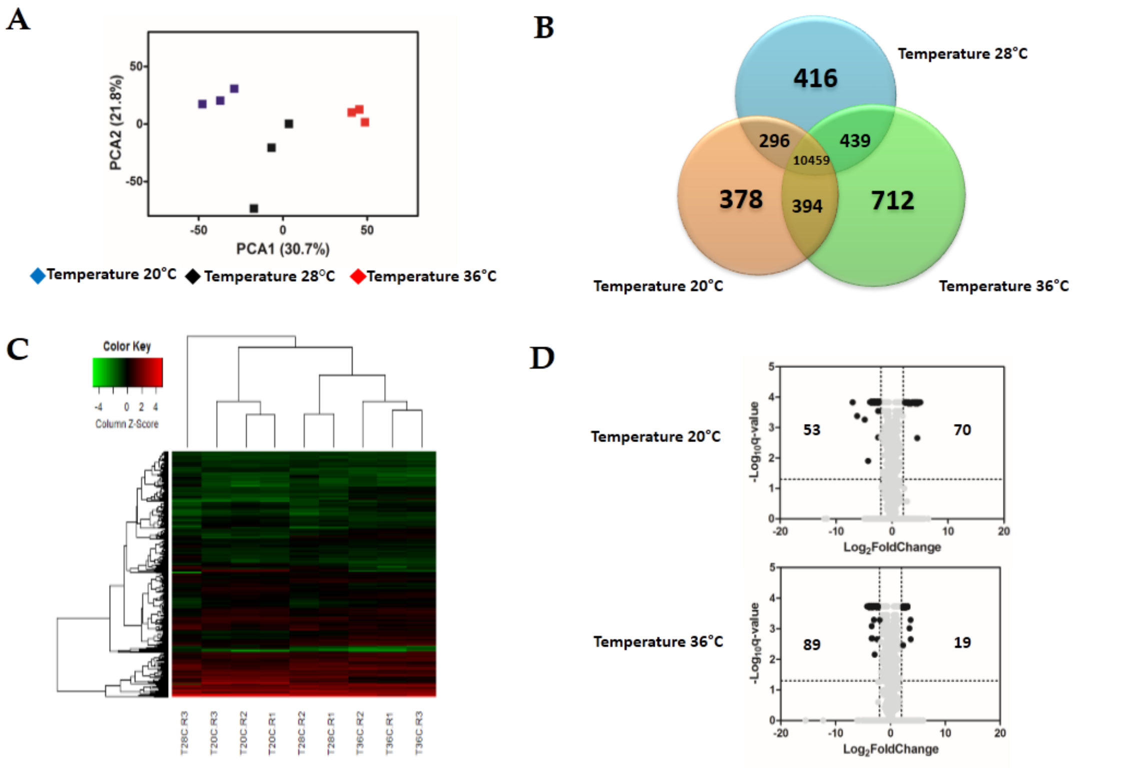

Supplementary Figure S1. Effect of the temperature in the expression profile of non-infected *A. aegypti.* **(A)** Principal Component Analysis (PCA) plot showing the global gene expression profiles. **(B)** Venn Diagram reporting the number of specific and shared genes. **(C)** Heatmap plot showing local differences (TC20R1: represents the replicate 1 for the temperature 20ºC). **(D)** Volcano plot representing the differential gene expression from RNAseq samples from *Aedes aegypti* exposed in three different constant temperatures (20ºC, 28ºC, 36ºC) at 24 hs.

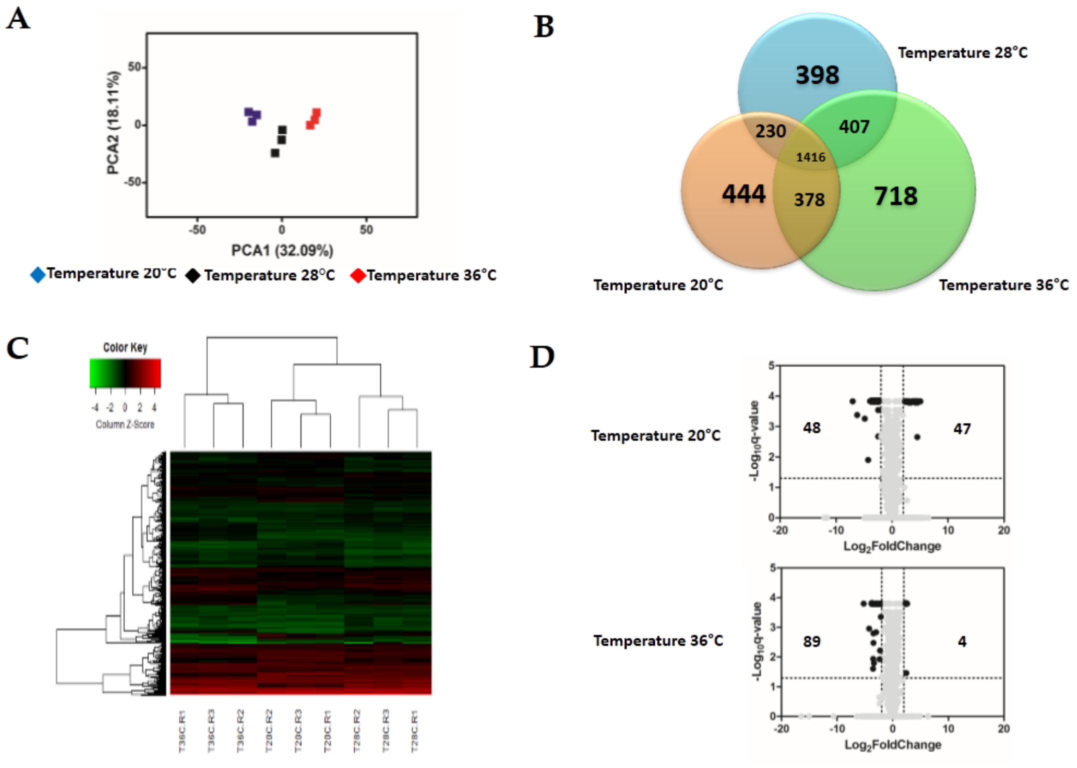

Supplementary Figure S2. Effect of the temperature in the expression profile of Zika-infected *A. aegypti.* **(A)** Principal Component Analysis (PCA) plot showing the global gene expression profiles. **(B)** Venn Diagram reporting the number of specific and shared genes. **(C)** Heatmap plot showing local differences (TC20R1: represents the replicate 1 for the temperature 20ºC). **(D)** Volcano plot representing the differential gene expression from RNAseq samples from infected *Aedes aegypti* exposed in three different constant temperatures (20ºC, 28ºC, 36ºC) at 24 hs.

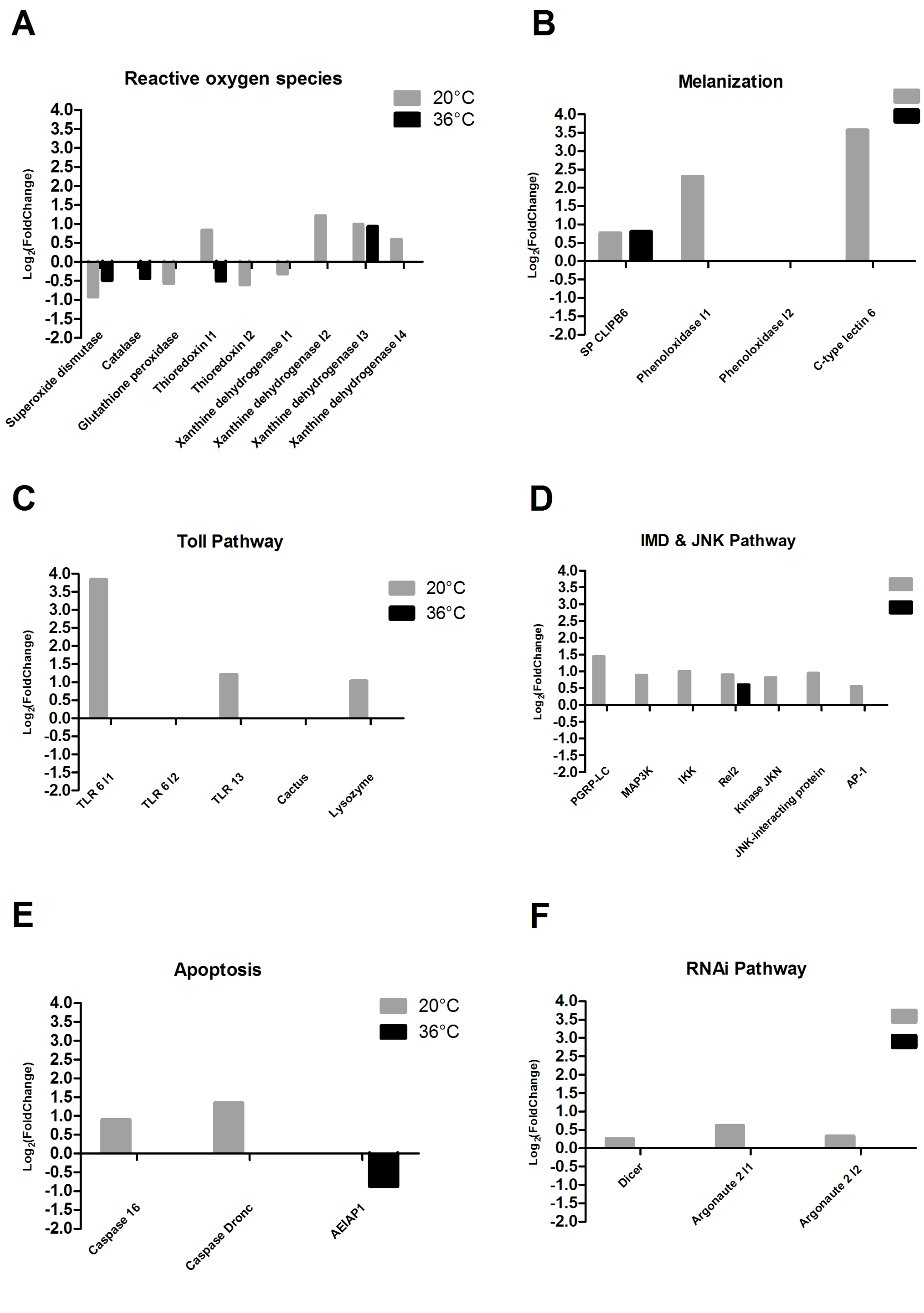

Supplementary Figure S3. Analysis of differentially expressed genes related to the immune response in uninfected mosquitoes maintained at 20°C and 36°C compared to those kept at 28°C 48 hours after blood-feeding (A) Reactive Oxygen Species; **(B)** Melanization; **(C)** Toll Pathway; **(D)** IMD & JNK Pathway; **(E)** Apoptosis; **(F)** RNAi Pathway.

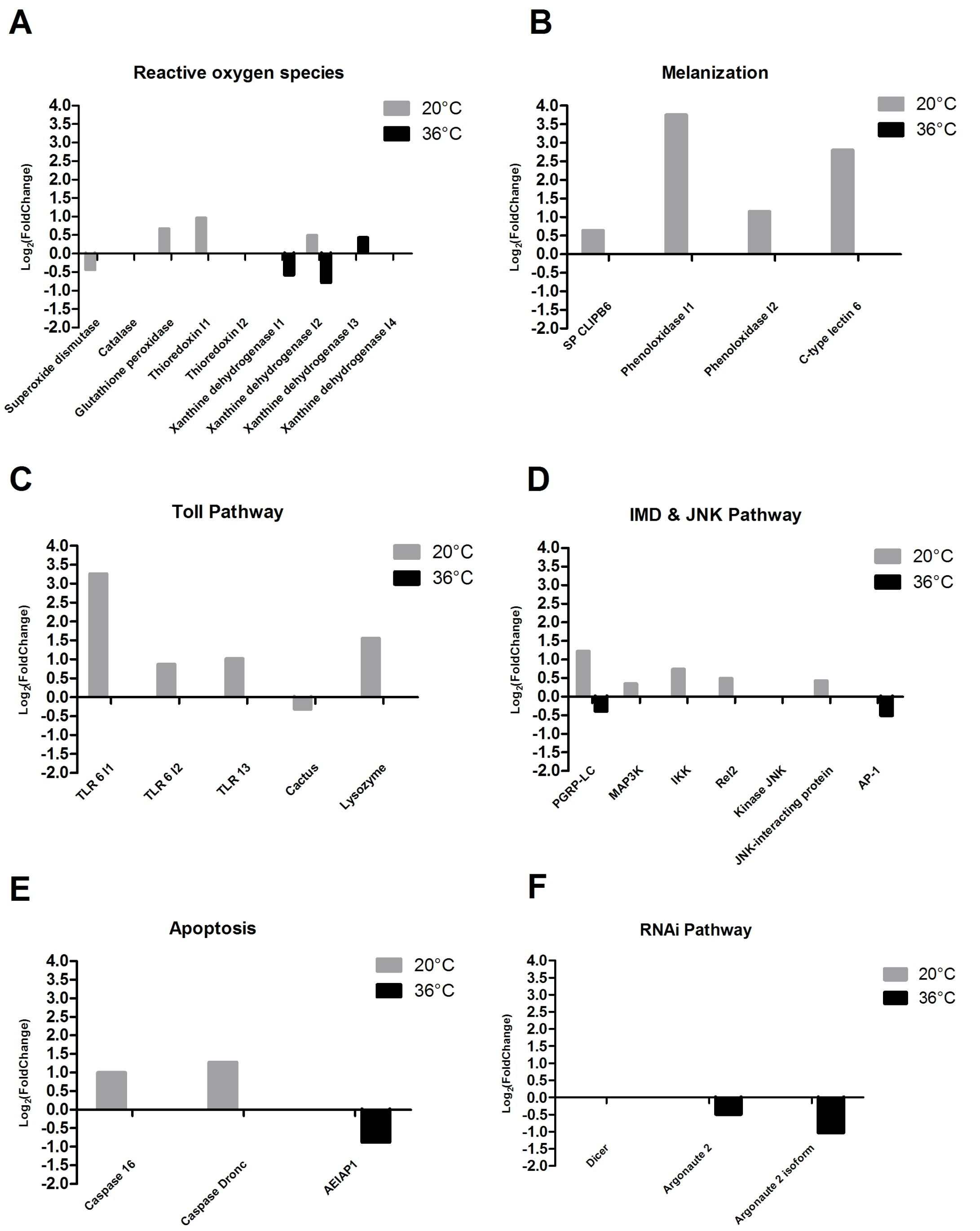

Supplementary Figure S4. Analysis of differentially expressed genes related to the immune response in ZIKV-infected mosquitoes maintained at 20°C and 36°C compared to those kept at 28°C 48 hours after blood-feeding (A) Reactive Oxygen Species; **(B)** Melanization; **(C)** Toll Pathway; **(D)** IMD & JNK Pathway; **(E)** Apoptosis; **(F)** RNAi Pathway.

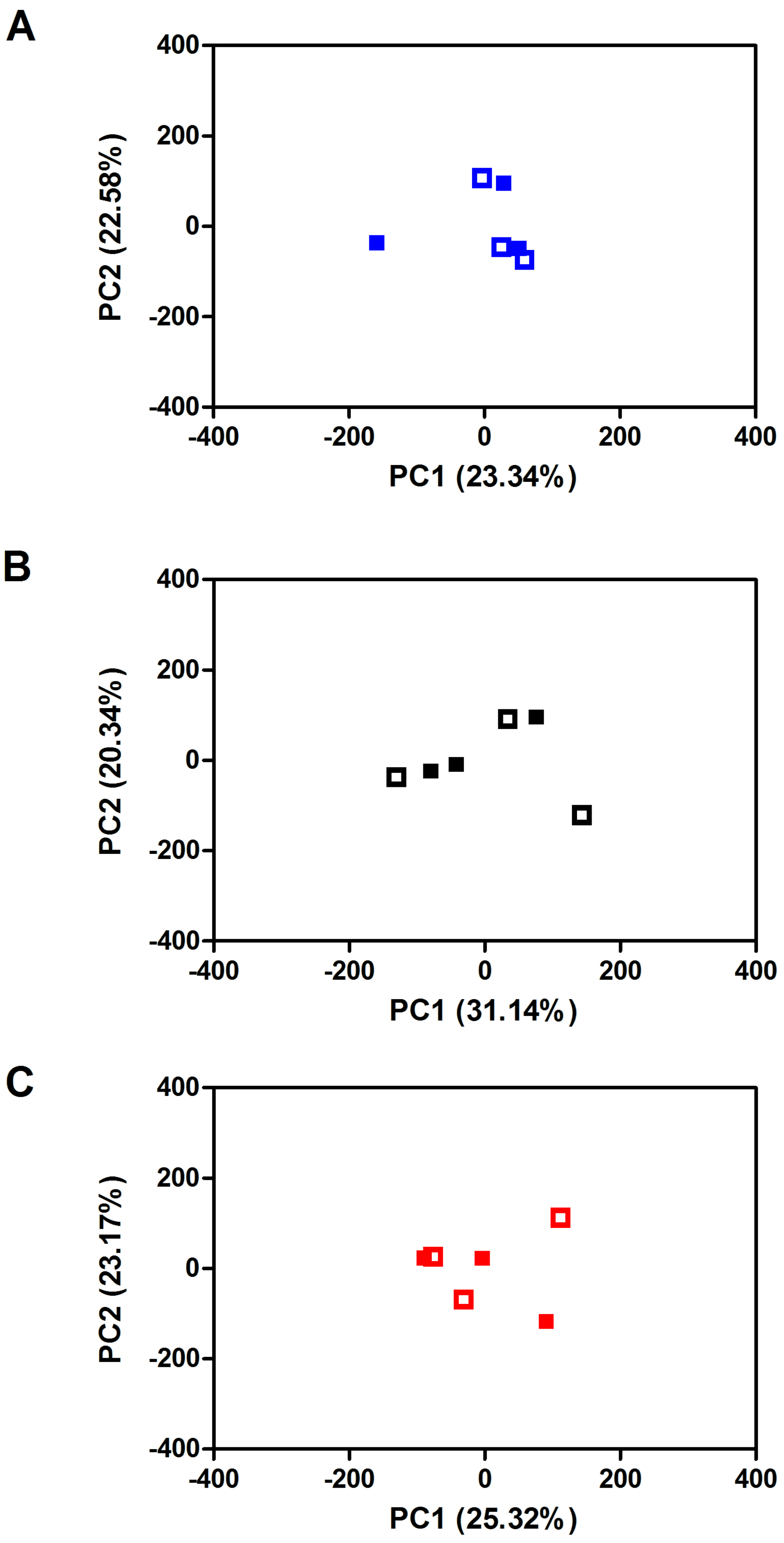

A
400
200
0
-200
-400
-400
-200
0
200
400
PC2 (22.58%)
PC1 (23.34%)
B
PC2 (20.34%)
PC1 (31.14%)
C
PC2 (23.17%)
PC1 (25.32%)

Supplementary Figure S5. The Principal Component Analysis (PCA) of the effect of the Zika infection in three different constant temperatures (A) 20ºC (B) 28ºC (C) 36ºC at 24hs. Open diamond: non-exposed mosquitoes. Closed diamond: ZIKV-exposed mosquitoes.

A

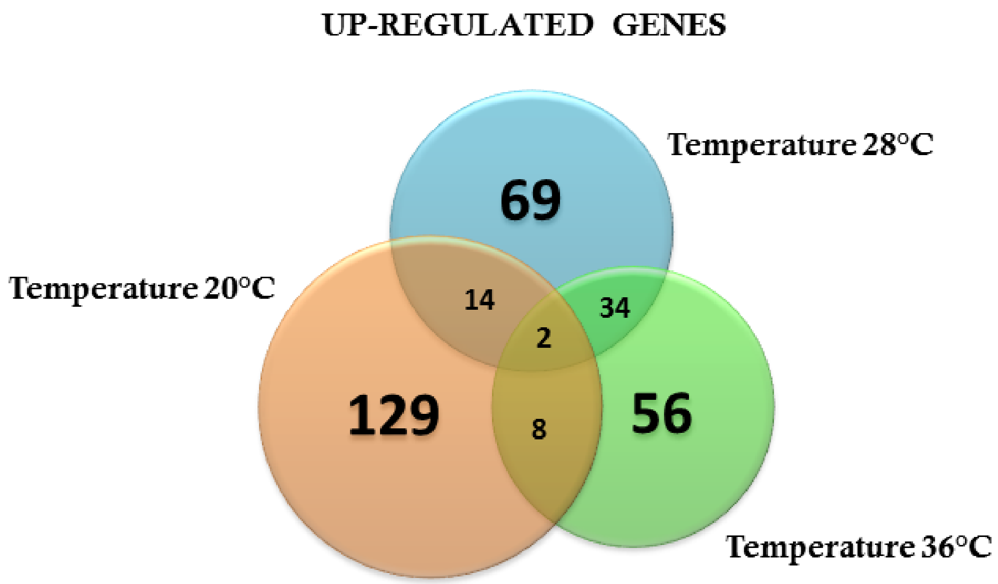

B

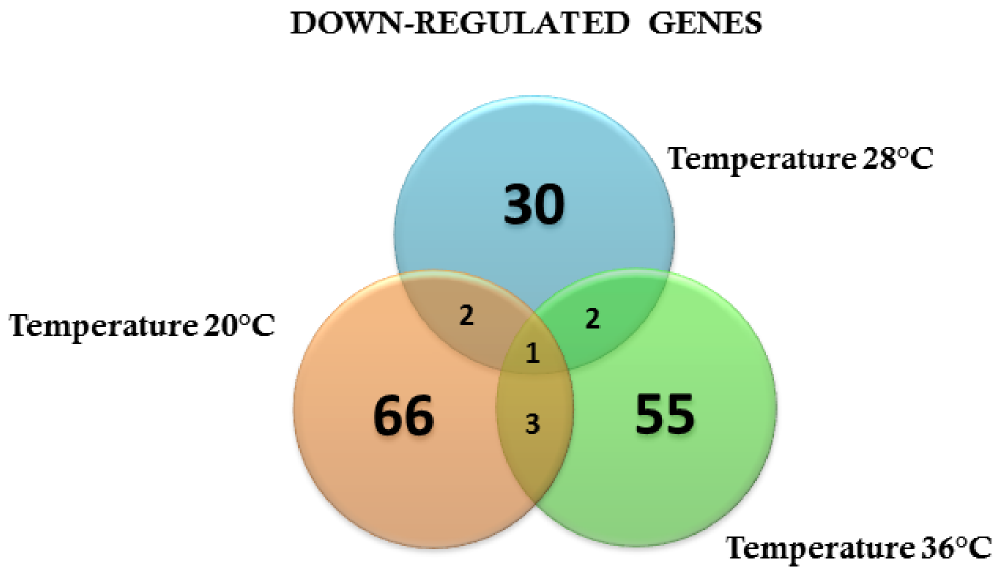

Supplementary Figure S6. Venn diagram representing the number of specific differentially expressed genes for Zika infected *A. aegypti* exposed in three different constant temperatures (20ºC, 28ºC, 36ºC) (A) Up regulated genes **(B)** Down regulated genes at 24hs.

Supplementary Table Captions

Supplementary Table S1. Differentially expressed genes in mosquitoes held at 20°C relative to those held at 28°C 24 and 48hs after uninfected control blood meal.

Supplementary Table S2. Differentially expressed genes in mosquitoes held at 36°C relative to those held at 28°C 24 and 48hs after uninfected control blood meal.

Supplementary Table S3. Differentially expressed genes involved in oxidative stress and innate immunity in mosquitoes held at 20°C and 36°C relative to those held at 28°C 48hs after uninfected control blood meal.

Supplementary Table S4. Differentially expressed genes involved in oxidative stress and innate immunity in mosquitoes held at 20°C and 36°C relative to those held at 28°C 48hs after ZIKV-positive blood meal.

Supplementary Table S5. Differentially expressed genes in mosquitoes held at 20°C relative to those held at 28°C 24 and 48hs after ZIKV-positive blood meal.

Supplementary Table S6. Differentially expressed genes in mosquitoes held at 36°C relative to those held at 28°C 24 and 48hs after ZIKV-positive blood meal.

Supplementary Table S7. Differentially expressed genes in mosquitoes 24 and 48 hours after ZIKV-positive blood meal relative to mosquitoes fed with uninfected blood held at 20°C.

Supplementary Table S8. Differentially expressed genes in mosquitoes 24 and 48 hours after ZIKV-positive blood meal relative to mosquitoes fed with uninfected blood held at 28°C.

Supplementary Table S9. Differentially expressed genes in mosquitoes 24 and 48 hours after ZIKV-positive blood meal relative to mosquitoes fed with uninfected blood held at 36°C.
